# Live and inactivated *Salmonella enterica* serovar Typhimurium stimulate similar but distinct transcriptome profiles in bovine macrophages and dendritic cells

**DOI:** 10.1186/s13567-016-0328-y

**Published:** 2016-03-22

**Authors:** Kirsty Jensen, Iain J. Gallagher, Anna Kaliszewska, Chen Zhang, Oluyinka Abejide, Maurice P. Gallagher, Dirk Werling, Elizabeth J. Glass

**Affiliations:** The Roslin Institute and Royal (Dick) School of Veterinary Studies, University of Edinburgh, Easter Bush, Edinburgh, EH25 9RG UK; Health and Exercise Research Group, University of Stirling, Cottrell Building, Stirling, FK9 4LA UK; Scotland’s Rural College, Easter Bush, Edinburgh, EH25 9RG UK; Institute of Cell Biology, University of Edinburgh, King’s Buildings, Mayfield Road, Edinburgh, EH9 3JR UK; Department of Pathology and Pathogen Biology, Royal Veterinary College, Hawkshead Lane, Hatfield, AL9 7TA UK

## Abstract

*Salmonella enterica* serovar Typhimurium (*S.* Typhimurium) is a major cause of gastroenteritis in cattle and humans. Dendritic cells (DC) and macrophages (Mø) are major players in early immunity to *Salmonella*, and their response could influence the course of infection. Therefore, the global transcriptional response of bovine monocyte-derived DC and Mø to stimulation with live and inactivated *S.* Typhimurium was compared. Both cell types mount a major response 2 h post infection, with a core common response conserved across cell-type and stimuli. However, three of the most affected pathways; inflammatory response, regulation of transcription and regulation of programmed cell death, exhibited cell-type and stimuli-specific differences. The expression of a subset of genes associated with these pathways was investigated further. The inflammatory response was greater in Mø than DC, in the number of genes and the enhanced expression of common genes, e.g., interleukin (IL) 1B and IL6, while the opposite pattern was observed with interferon gamma. Furthermore, a large proportion of the investigated genes exhibited stimuli-specific differential expression, e.g., Mediterranean fever. Two-thirds of the investigated transcription factors were significantly differentially expressed in response to live and inactivated *Salmonella*. Therefore the transcriptional responses of bovine DC and Mø during early *S.* Typhimurium infection are similar but distinct, potentially due to the overall function of these cell-types. The differences in response of the host cell will influence down-stream events, thus impacting on the subsequent immune response generated during the course of the infection.

**Electronic supplementary material:**

The online version of this article (doi:10.1186/s13567-016-0328-y) contains supplementary material, which is available to authorized users.

## Introduction

*Salmonella enterica* is one of the major causes of food-borne disease worldwide. Over 2500 serovars of *S. enterica* have been identified, which exhibit differences in host-specificity and disease outcome. *S. enterica* serovars Typhi (*S.* Typhi) and Dublin (*S.* Dublin) exhibit restricted host specificity, principally causing systemic disease in humans and cattle respectively. In contrast, *S. enterica* serovar Typhimurium (*S.* Typhimurium) infects a broad range of unrelated host species, including cattle and humans, causing gastroenteritis. *S.* Typhimurium rarely causes systemic disease, except in mice, where the disease mimics Typhoid fever in humans caused by *S.* Typhi [1]. In cattle, *S.* Typhimurium infection most commonly causes clinical disease in calves between 2 and 6 weeks of age. Symptoms mirror those observed in humans and include diarrhoea, anorexia and pyrexia within 12–48 h of infection [1]. Infected cattle can excrete 10^8^ cfu *Salmonella* per gram of faeces and therefore are a major source of contamination and a potential risk to other cattle and humans.

*S.* Typhimurium is one of the major serovars causing disease in cattle in the US and UK [2, 3]. A large proportion of *S.* Typhimurium infections in the UK involve strain DT104, which contains a phage encoding for resistance to most antimicrobials [3, 4]. Therefore, alternative methods of control are needed, the development of which requires further understanding of the host-pathogen interactions occurring during infection. The only vaccine licenced in the UK against *Salmonella* infection in cattle consists of inactivated *S.* Dublin and *S.* Typhimurium. This vaccine does not induce sterile immunity but decreases the risk of disease and reduces shedding and is principally used during outbreaks [5].

Four hours after experimental oral challenge of calves, *S.* Typhimurium was found to have traversed the ileal epithelium and was detected within phagocytes in the lamina propria [6]. To infect non-phagocytic epithelial cells *S.* Typhimurium employs genes within a region of the genome termed the *Salmonella* pathogenicity island 1 (SPI-1), which encodes a type three secretion system (T3SS) that injects SPI-1 encoded effector proteins into the host cell cytosol, stimulating cytoskeletal alterations, leading to membrane ruffling and internalization of *Salmonella* by pinocytosis [7]. Some *Salmonella* then traverse to the basolateral side of the epithelial cell and exit via exocytosis into the interstitial space before being rapidly engulfed by phagocytes [8].

The phagocytes that engulf *Salmonella* in the lamina propria include neutrophils, which flood into the area in response to chemoattractants released by infected epithelial cells. In addition, *Salmonella* is taken up by resident antigen presenting cells (APC); macrophages (Mø) and dendritic cells (DC). *Salmonella* survives and replicates in Mø, which requires genes encoded within the *Salmonella* pathogenicity island 2 (SPI-2) [7]. In contrast, *S.* Typhimurium only persists in murine DC without replicating [9, 10]. The response of bovine monocyte-derived Mø and DC to in vitro *S.* Typhimurium infection was found to differ [11]. Transcripts of interleukin (IL) 12 and colony stimulating factor (CSF) 2 were up-regulated in DC, whilst IL10 was only up-regulated in Mø. In agreement with this pattern, IL12 and IL10 protein release was greater in DC and Mø, respectively, in response to heat-inactivated *S.* Dublin [12]. The cell-specific release of different cytokines would alter the signalling to other immune cells, thus potentially affecting not only the innate, but also the development of the adaptive immune response at the site of infection. In turn, this may influence the course of the *Salmonella* infection.

To investigate early events which might lead to these differences we have compared the global transcriptional response of bovine monocyte-derived Mø and DC to early *S.* Typhimurium infection. *S.* Typhimurium infects Mø and DC in the lamina propria once the bacteria has passed across the epithelial layer. The bacteria can be internalized by these phagocytes by phagocytosis or SPI-1 mediated pinocytosis [13] and it is unclear which mechanism predominates in the lamina propria. Irrespective of the mode of entry, *Salmonella* survive within these cells in *Salmonella* containing vacuoles (SCVs) [6], which are fully mature approximately 1 h post infection [14] and *Salmonella* starts to replicate 3–4 h post infection [15]. We investigated the transcriptional response of Mø and DC at a time between these two events, 2 h post infection, when *S.* Typhimurium is establishing a niche inside the cell. Furthermore, in an attempt to separate out the transcriptional response induced by the detection of PAMPs, e.g., LPS, flagellin, from that induced by the interaction with *S.* Typhimurium effector proteins, we compared the response of Mø and DC to live and inactivated *S.* Typhimurium. This may lead to insights into why inactivated *Salmonella* vaccines are relatively ineffective. Overall we found a similar transcriptional response by Mø and DC to live and inactivated *S.* Typhimurium. However, there were quantitative differences in the expression of a large proportion of the genes investigated at this early time point of infection, which may alter events down-stream during infection in both the infected cells and other immune cells.

## Materials and methods

### Animals

Cells were isolated from female Holstein–Friesian cattle (*Bos taurus*) maintained at The Roslin Institute, University of Edinburgh, UK. These Holstein–Friesian cattle were between 6 months to 5 years of age and kept on pasture. All experimental protocols were authorized under the UK Animals (Scientific Procedures) Act, 1986. In addition, The Roslin Institute’s Animal Welfare and Ethics Committee (AWEC) ensure compliance with all relevant legislation and promote the adoption and developments of the 3Rs.

### Culture of monocyte-derived Mø

The bovine Mø used for the microarray and RT-qPCR studies were generated using different methods and derived from cells isolated from different animals. For the former, Mø were generated from peripheral blood essentially as described previously [16]. Blood samples were collected aseptically by jugular venipuncture into acid citrate dextrose (ACD) and buffy coats were separated by centrifugation. These cells were washed three times with citrate buffer (30 mM citric acid, 0.6% NaCl, 3 mM KCl, 4.8 mM glucose), to remove fibrinogen. Peripheral blood mononuclear cells (PBMC) were separated by density gradient centrifugation on Lymphoprep (Axis-Shield) and resuspended at 5 × 10^6^ cells/mL in Iscove’s modified Dulbecco’s medium (IMDM) (Invitrogen) supplemented with GlutaMax™ (Invitrogen), 25 mM HEPES, 100 IU/mL penicillin, 100 μg/mL streptomycin, 10 mM sodium pyruvate, 1% minimum essential medium (MEM) vitamins (Invitrogen), 1% non-essential amino acids (Invitrogen), 50 μM β-mercaptoethanol, 10 µg/mL gentamicin and 20% heat-inactivated foetal bovine serum (FBS) (Biosera). The PBMC were cultured in non-adherent Teflon bags for 7 days at 37 °C in 5% CO_2_, during which time the monocytes differentiated into Mø [16]. Cells were then resuspended in fresh medium supplemented as above, except that the FBS concentration was reduced to 2%. Mø were purified by selective adherence for at least 6 h and non-adherent cells were thoroughly washed off. The purity of the Mø population was assessed by flow cytometric analysis using anti-human CD14, anti-bovine SIRPA, anti-bovine CD3 and anti-bovine CD21 antibodies using a CyAn ADP flow cytometer (Beckman Coulter) (data not shown) and exceeded 90%. The maturation of monocytes to Mø was confirmed by morphological examination and by RT-PCR analysis of selected Mø markers, e.g., CD16 and CD180, which showed strong expression in the generated Mø (data not shown).

The Mø used in the validation studies were generated as previously described [17] from peripheral PBMC cultured in flasks for 5 days and purified by adherence. Flow cytometric analysis using anti-human CD14, anti-bovine SIRPA, anti-bovine CD3 and anti-bovine CD21 antibodies confirmed that the Mø purity exceeded 95% (Additional file 1). The Mø were replated at 3 × 10^5^ cells/mL in RPMI-1640 medium supplemented with 20% FBS and cultured for 48 h before stimulation to ensure that the cells had returned to a resting state.

### Culture of monocyte-derived DC

The bovine DC used for the microarray and RT-qPCR studies were generated in a similar way but were derived from different animals. Blood was collected aseptically into ACD and PBMC were separated by density gradient centrifugation on Lymphoprep (Axis-Shield). CD14^+^ cells were separated by positive selection using microbeads conjugated with an anti-human CD14 antibody and the MACS system (Miltenyi Biotec). The purity of the resulting CD14^+^ cells was assessed by flow cytometry using an anti-bovine SIRPA monoclonal antibody (AbD Serotec) and exceeded 95% (data not shown). The cells were resuspended at 1 × 10^6^ cells/mL in RPMI-1640 (Invitrogen) medium supplemented with 10% FBS, containing either recombinant bovine IL4 and recombinant CSF2 (kindly provided by Dr Gary Entrician, Moredun Research Institute, UK), at pre-determined optimal concentrations, or bovine DC growth kit (AbD Serotec). The cells were cultured at 37 °C in the humidified atmosphere of 5% CO_2_. After 5 days of culture the DC had acquired the morphology and phenotype of previously described monocyte-derived DC [18]. To confirm the cell purity flow cytometric analysis using anti-human CD14, anti-bovine SIRPA, anti-bovine CD3 and anti-bovine CD21 antibodies was performed (Additional file 1). In addition, the generation of DC was confirmed by RT-PCR analysis of selected DC markers, e.g., CD1B, which showed strong expression in the generated DC (data not shown). The DC were replated at 3 × 10^5^ cells/mL in RPMI-1640 medium supplemented with 10% FBS and DC growth kit and cultured for 48 h before stimulation to ensure that the cells had returned to a resting state.

The expression of cell surface molecules and DC/Mø associated transcripts was compared between the Mø and DC cultures (Additional file 1). These analyses confirmed that the culture methodologies employed had resulted in the generation of distinct cell populations.

### Infection and stimulation of the cells with *Salmonella*

*S.* Typhimurium strain SL1344, verified by API-20E test (bioMerieux) (data not shown), was grown overnight at 37 °C in Luria–Bertani (LB) broth. The bacteria were washed once with phosphate buffered saline (PBS) and resuspended in fresh PBS. The bacteria were inactivated either by heating for 40 min at 80 °C or alternatively by treatment with 1% paraformaldehyde (PFA) for 1 h. The bacteria were then washed three times with PBS and resuspended in fresh PBS. Successful inactivation of bacteria was verified by the lack of colonies following overnight incubation on LB agar plates. Inactivated and live bacteria were added to the Mø and DC cultures to give a multiplicity of infection (MOI) of 50. Preliminary work using GFP-expressing *S.* Typhimurium SL1344 confirmed uptake of bacteria by over 90% Mø and DC using this experimental design (data not shown). The inocula numbers were confirmed by colony counts on LB agar plates. Control, resting samples were left untreated. After incubation for 2 h the APC were harvested and the RNA extracted. For addition studies RNA was extracted from Mø 0.5, 1, 2 and 3 h post stimulation. In addition, Mø were incubated for 8 h post stimulation and the supernatants used for ELISA.

### Microarray experimental design

Mø and DC were prepared in parallel from six cattle. Each cell sample was cultured for 2 h at 37 °C in medium alone, exposed to live *S.* Typhimurium or exposed to heat-inactivated *S.* Typhimurium. Therefore a total of 36 samples were collected. The Affymetrix GeneChip^®^ Bovine Genome Array, which measures the expression levels of over 23000 transcripts, was used to perform the transcriptomic experiment. The microarray data is publicly available at ArrayExpress and has the accession number E-TABM-878.

### RNA preparation and microarray hybridization

RNA was extracted from Mø and DC using the RNeasy MiniKit (Qiagen) according to manufacturer’s instructions. The RNA concentration was measured using a NanoDrop ND100 Spectrophotometer and RNA quality was checked by Agilent 2100 Bioanalyzer. The RNA integrity number (RIN) exceeded 7 for all samples. Samples were concentrated using the Linear Acrylamide Precipitation System (Ambion). Two-cycle amplification and labelling of the RNA was performed using the GeneChip Expression 3′ Amplification Two-Cycle Target Labelling and Control Reagents Kit (Affymetrix), the IVT cRNA Cleanup Kit (Affymetrix) and the MEGAscript High Yield Transcription Kit (Ambion) according to the manufacturers’ instructions. The resulting copy RNA (cRNA) concentration and quality were measured by NanoDrop and Agilent Bioanalyzer respectively. 20 µg biotinylated cRNA was fragmented using 5 × Fragmentation Buffer (Affymetrix) and hybridized onto the Affymetrix GeneChip^®^ Bovine Genome Array.

The hybridized microarrays were scanned using the Affymetrix scanner and the Affymetrix Genechip Operating software (GCOS) was used for the analysis of gene expression and expression clustering. Images were quantified with the command console software. Two slides failed the preliminary quality control (QC) and subsequent analysis, including principal component analysis (Additional file 2), identified issues with a number of other slides, which were removed from the analysis. As a result, four biological replicates were included in the analysis for each cell-type/condition.

### Statistical and bioinformatic analysis of the microarray data

The microarray data were analysed in R (version 2.14.1) using the bioconductor 2.9 libraries [19]. Briefly, after QC and normalization using the robust multiarray average (RMA) algorithm [20] MAS5. Absent calls were used to remove any probe-set absent across all the arrays. The paired sample analysis implemented in the significance analysis of microarrays (SAM) algorithm [21] was used to identify differentially regulated genes by comparing live and inactivated *S.* Typhimurium treated samples with the uninfected controls. Genes with false discovery rate (FDR) [22] less than 5% and absolute fold changes greater than 1.8 were considered significantly differently expressed. The Affymetrix Bovine Annotation (version 32) was used to annotate the differentially expressed probe-sets. If there was no designated description, the probe-sets were annotated manually using the consensus sequences, provided by Affymetrix, which had been used to design the probe-sets. The consensus sequences were blasted against the bovine genome (UMD3.1) and sequence databases e.g., NCBI.

The Database for annotation, visualization and integrated discovery (DAVID) [23, 24] was used to perform functional annotation analysis. Initially the analysis was carried out using the Affymetrix probe ID and setting the microarray as the background. However, due to the relative paucity of gene ontology (GO) annotation of bovine genes, the gene enrichment analysis was repeated using the HUGO approved gene symbols and the human genome was set as the background. The analysis was carried out to identify over-represented Biological Process GO terms.

### PCR analysis of unannotated transcripts

RT-PCR analysis was carried out to investigate if selected unannotated transcripts represent novel splice variants. Oligonucleotides were designed to amplify products spanning the exonic sequence of neighbouring genes and the consensus sequence for the unannotated probe-sets provided by Affymetrix. One set of oligonucleotides was designed to investigate Bt.19462, the forward primer aligns to exon 3 of chemokine (C–C motif) ligand 5 (CCL5) (5′-TGCTGTGAAAGACCCTCAGT-3′) and the reverse primer aligns to the consensus sequence of Bt.19462 (5′-GCAGATTGAAGATGGAAGAGAA-3′). Two sets of oligonucleotides were designed to investigate Bt.17514; the first set consists of a forward primer which aligns to exon 1 of tumour necrosis factor, alpha-induced protein (TNFAIP) 3 (5′-CGAGAAGTCAGGAGGCTTG-3′) and the reverse primer aligns to the consensus sequence of Bt.17514 (5′-ACAACACCACCACCACCAC-3′). The second oligonucleotide pair consists of a forward primer that aligns to the consensus sequence of Bt.17514 (5′-GAGGATATGAGACTGCGGTGA-3′) and a reverse primer that aligns to exon 2 of TNFAIP3 (5′-CGGGTGTCGTAGCAAAGC-3′).

The oligonucleotide pairs were used to amplify products from bovine monocyte-derived Mø cDNA samples generated from total RNA using oligo(dT) primer and GoScript (Promega) according to the manufacturer’s instructions. The products were amplified using ABgene Taq polymerase (Thermo Scientific) and purified using QIAquick Gel Extraction Kit following the manufacturers’ instructions. Purified PCR fragments were cloned into pGEM-T easy vector (Promega) and the resulting plasmids purified using the Qiaprep miniprep kit (Qiagen) following the manufacturers’ instructions. The purified plasmids were sequenced using Big Dye Sequencing and T7 and SP6 oligonucleotides (Edinburgh Genomics).

### RT-quantitative PCR (RT-qPCR) analysis of mRNA levels

The mRNA levels of the selected transcripts were quantified by RT-qPCR using different biological samples than those used for the microarray experiment. First strand cDNA was generated as described above and the qPCR was carried out using the Brilliant III ultra-fast SYBR Green Mastermix kit (Agilent). Oligonucleotides were designed for each gene using Primer3 [25, 26] and Netprimer (Biosoft International) software (Additional file 3). PCR products generated with each oligonucleotide set were sequenced to ensure amplification of the correct transcript. Reactions were carried out in 10 μL volumes containing; 1 × SYBR Green Master mix and reference dye, 0.5 μL forward and reverse primers at predetermined optimal concentrations and 2.5 μL cDNA diluted at 1:20 for all genes. Amplification and detection of products was carried out using a Mx3000P PCR machine (Stratagene) with the following cycle profile: 95 °C for 3 min followed by 50 cycles of 95 °C for 10 s and 60 °C for 22 s. The detection of a single product was verified by dissociation curve analysis. Each PCR experiment was carried out in triplicate and contained several non-template controls and a log_10_ dilution series of the representative standard. The relative quantities of mRNA were calculated using the method described by [27]. The results for each target gene were normalized against the results for squamous cell carcinoma antigen recognized by T cells (SART1). SART1 was among a number of genes selected from analysis of the microarray data as being constitutively and moderately expressed in all the biological samples (data not shown). Further RT-qPCR analysis revealed that SART1 was the most suitable reference gene to use from the selected genes and other normalization genes frequently used in our laboratory (data not shown).

### Knock-down of target genes by siRNA

Purified bovine monocyte-derived Mø were resuspended at 3×10^5^ cells/mL in RPMI-1640 medium, supplemented with 20% FBS, dispensed into 12 well plates and cultured for 48 h before transfection of siRNA. siRNA duplexes specific for NLR family, pyrin domain containing 3 (NLRP3) (target sequence GTGTATATCTTCTTCCTCT) and Mediterranean Fever (MEFV) (target sequence GTTGCTTAATAAATCCTTA, described previously [17]) were designed and supplied by Sigma–Aldrich. The Mø were transfected with siRNA following the manufacturer’s protocol, initially generating a mix of 3 μL Lipofectamine RNAiMAX (Invitrogen) and 3 μL 20 μM siRNA in 200 μL Opti-MEM I reduced serum medium (Invitrogen). After 20 min incubation at room temperature the siRNA/Lipofectamine RNAiMAX mix was added to the Mø in 1 mL RPMI-1640 medium, supplemented with 20% FBS, giving a final concentration of 50 nM siRNA. Additional controls included in each experiment were Mø treated with Lipofectamine RNAiMAX only (transfection control) and untreated Mø (negative control). In addition, the AllStars negative control siRNA (Qiagen), which does not share homology with any known mammalian gene, was used as a non-target siRNA control. After 24 h the medium was replaced with fresh medium to remove the residual siRNA/Lipofectamine RNAiMAX mix. After a further 24 h cells were infected with live *S.* Typhimurium as described above.

### Enzyme-linked immunosorbent assays (ELISA)

Supernatants were collected from Mø stimulated with live and PFA-inactivated *S.* Typhimurium for 8 h. Tumour necrosis factor (TNF) and IL1B protein levels in the supernatants were quantified by Bovine TNFα DuoSet ELISA (R&D Systems) and Bovine IL1β Screening Kit (Thermo Scientific Pierce) respectively, following the manufacturers’ instructions. Interferon gamma (IFNG) protein was quantified by ELISA using the mouse anti-bovine IFNG antibodies MCA2112 and MCA1783B (AbD Serotec) as coating and detecting antibodies respectively. Both antibodies were used at 2 μg/mL final concentration. The same protocol and buffers were used in this ELISA and the IL1B ELISA. The concentration of IFNG was determined using a standard curve of recombinant bovine IFNG (kindly provided by Dr. Jayne Hope, The Roslin Institute). All ELISAs were carried out using Nunc-Immuno Maxisorp 96 well plates, with all samples and standards in triplicate. The plates were read at 450 nm, with the reference values at a wavelength of 550 nm subtracted, using a Synergy HT plate reader (BioTek).

### Statistical analysis

All statistical analysis was carried out using Minitab version 17. ELISA results were analysed by *t* test. The RT-qPCR data were transformed on the log_2_ scale before statistical analyses to stabilize the variance. Changes in gene expression compared to resting cells and differences in the response to live and inactivated *Salmonella* were analysed by *t* test with *P* value correction, using the Benjamini–Hochberg procedure [28], to adjust for multiple testing. Comparison of the response of DC and Mø was analysed by General Linear Model (GLM), fitting animals as a random effect, with cell-type (DC and Mø) and condition (unstimulated, live *Salmonella* infection and inactivated *Salmonella* stimulation) as fixed effects. Subsequent Fisher’s tests, with Benjamini–Hochberg adjustment of *P* values, were used to identify significant differences between cell types and conditions, as well as the interaction between cell-type and condition. Similarly, the time course data was analysed by GLM to allow repeated measures analysis.

## Results

### Analysis of the microarray data

The response of bovine monocyte-derived DC and Mø to stimulation with live and inactivated *S.* Typhimurium for 2 h was investigated using the Affymetrix Bovine Genome Array. Differentially expressed genes were identified for each sample group; DC infected with live *S.* Typhimurium (DC_L), DC stimulated with inactivated *S.* Typhimurium (DC_D), Mø infected with live *S.* Typhimurium (Mø_L) and Mø stimulated with inactivated *S.* Typhimurium (Mø_D), by comparison with expression levels in the relevant uninfected samples (FDR ≤ 5%, fold change ≥1.8). The differentially expressed genes are summarized in Table 1 and listed in full in Additional file 4. The greatest change in gene expression was observed in Mø_L, with 221 probe-sets representing 193 differentially expressed genes being identified. Of these, seven probe-sets represent transcripts that could not be annotated. Live and inactivated *S.* Typhimurium elicited a lower transcriptional response in DC than Mø. Interestingly, there was a considerable bias towards up-regulated genes in all the gene lists. This was greater than 90% in DC_L, DC_D and Mø_D. Only Mø_L contained a higher proportion of down-regulated genes, although there was still a bias for up-regulated genes, with only 29% being repressed.

**Table 1:** **Summary of the differentially expressed genes identified by analysis of the microarray results**

Gene	**DC_L**	**DC_D**	**Mø_L**	**Mø_D**
No. probe-sets	126	156	221	230
No. transcripts	106	128	193	183
No. up-regulated genes	98	123	137	173
No. down-regulated genes	8 (7.5%)	5 (3.9%)	56 (29%)	10 (5.5%)
No. unannotated transcripts	7	9	12	13

The table summarizes the number of differentially expressed transcripts (FDR < 0.05, fold change >1.8) identified for DC treated with live (DC_L) and inactivated (DC_D) *S.* Typhimurium and Mø treated with live (Mø _L) and inactivated (Mø _D) *S.* Typhimurium compared to the uninfected DC or Mø samples.

All four gene lists contained genes that were represented by more than one probe-set, e.g., CASP8 and FADD-like apoptosis regulator (CFLAR) was represented by four probe-sets. Both the direction and magnitude of the differential expression were in agreement across the replicate probe-sets. The greatest fold up-regulation was observed in the Mø response to heat-inactivated bacteria, with 33.7-fold up-regulation of IL6. The observed down-regulation of gene expression did not reach that magnitude of change, the greatest down-regulation was observed in Mø in response to live infection, with a 4.0-fold decrease in thioredoxin interacting protein (TXNIP) expression.

**Figure 1:**
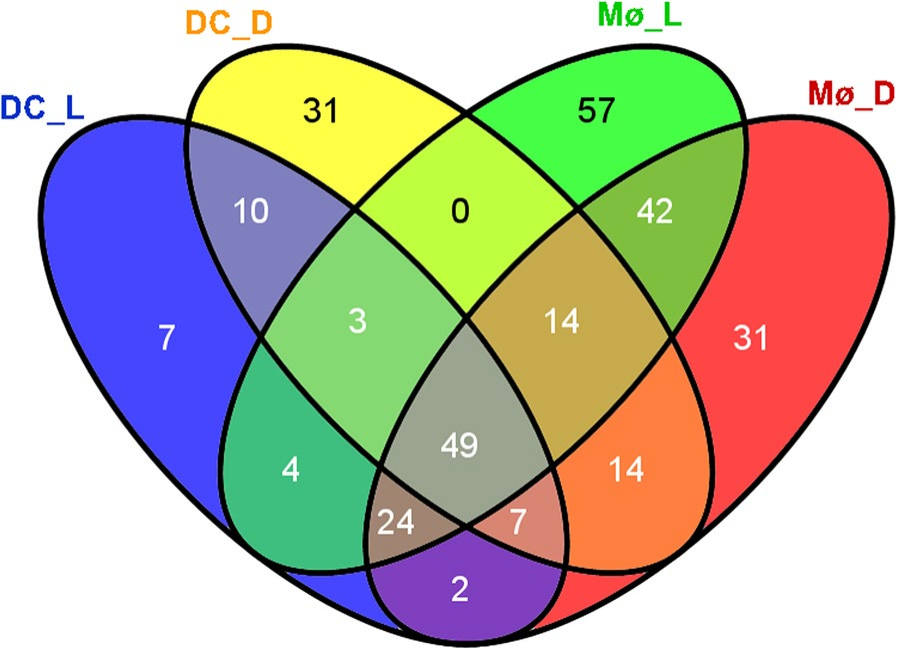
**Venn diagram illustrating the similarities and differences in the differentially expressed gene lists.** Venn diagram generated using Venny [64] illustrating the overlap between differentially expressed genes identified in DC infected with live *S.* Typhimurium (DC_L), DC stimulated with inactivated *S.* Typhimurium (DC_D), Mø infected with live *S.* Typhimurium (Mø_L) and Mø stimulated with inactivated *S.* Typhimurium (Mø_D).

A Venn diagram was used to investigate similarities and differences between the gene lists (Figure 1). The diagram illustrated that there was considerable overlap between the lists, however there were differentially expressed genes that were unique to each list, e.g., FBJ murine osteosarcoma viral oncogene homolog (FOS) and inhibitor of DNA binding 1, dominant negative helix-loop-helix protein (ID1) were among the 31 genes unique to the Mø_D gene list. In addition to these gene list specific genes, there were also genes that exhibited cell-type specific differential expression, e.g., activating transcription factor 3 (ATF3) and IL10 were only present in the Mø_D and Mø_L gene lists and nuclear receptor subfamily 4, group A, member 2 (NR4A2) was only present in DC_L and DC_D gene lists. Furthermore, analysis of the microarray data identified genes which were differentially expressed only in response to live *S.* Typhimurium, e.g., TXNIP, or heat-inactivated *S.* Typhimurium, e.g., FBJ murine osteosarcoma viral oncogene homolog B (FOSB).

#### Common transcriptional response

The Venn diagram (Figure 1) illustrated that 49 genes were differentially expressed in all four gene lists (Additional file 5) and therefore represent a common transcriptional response. Of these only one, paraneoplastic antigen MA1 (PNMA1), was down-regulated. CSF2 was consistently amongst the most up-regulated genes across all four gene lists. There was considerable variation in the level of differential expression observed in Mø and DC, the most extreme example of this is IL6, which was up-regulated by much higher levels in Mø, 17.7-fold and 33.7-fold in Mø_L and Mø_D respectively, compared to DC, 2.2-fold and 5.7-fold in DC_L and DC_D respectively. In addition, 14 of the common response genes (29%) exhibit more than 1.5-fold greater up-regulation in DC and/or Mø in response to inactivated *S.* Typhimurium compared to live bacteria. Again, IL6 is an example of this expression pattern.

**Figure 2:**
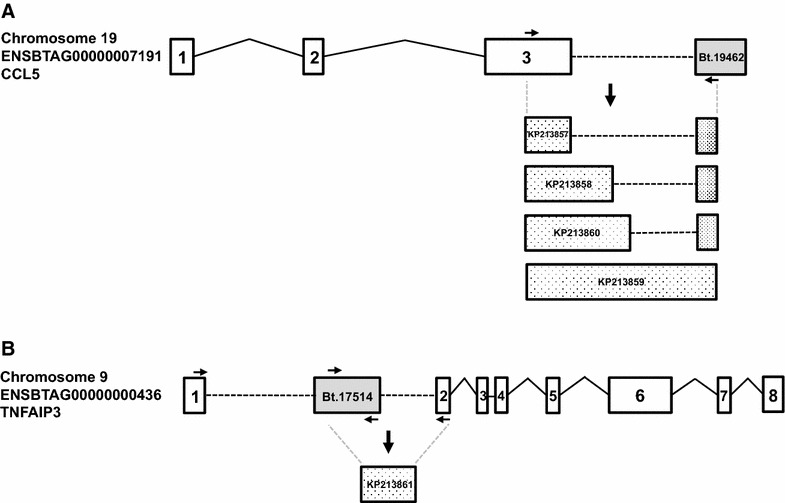
**Schematic diagram illustrating the alignment of unannotated sequences and nearby genes.** Gene structure of bovine **A** chemokine (C–C motif) ligand 5 (CCL5) and **B** tumour necrosis factor, alpha-induced protein 3 (TNFAIP3) and the position of the unannotated sequences Bt.19462 and Bt.17514 respectively. Open boxes denote known gene exons and grey boxes represent unannotated sequence. Solid lines denote intronic sequence. Dotted lines indicate predicted introns to accommodate unannotated sequences. Arrows illustrate the position of primers used to investigate if the unannotated transcripts represent previously unknown splice variants. Stippled boxes illustrate the sequenced novel transcripts and contain the relevant accession number.

Five unannotated transcripts were up-regulated in all four gene lists. Further analysis of these, by aligning to the bovine genome the consensus sequence used for probe-set design by Affymetrix, revealed that they all align either to intronic regions or immediately down-stream of known genes, the majority of which are associated with the immune response, e.g., v-rel avian reticuloendotheliosis viral oncogene homolog (REL), and apoptosis, e.g., BCL2-related protein A1 (BCL2A1). Two of the neighbouring genes: REL and TNFAIP3, are not represented by any annotated probe-set on the Affymetrix bovine microarray. However, the other three are represented. The Affymetrix probe-set Bt.19462 identifies a sequence immediately down-stream of CCL5 (Figure 2A), which is included in the list of common response genes. BCL2A1 is present in the DC_D gene list and RasGEF domain family, member 1B (RASGEF1B) is present in three of the differentially expressed gene lists (Additional file 4). To investigate if the unannotated transcripts represent previously unknown splice variants, oligonucleotides were designed to amplify transcripts spanning the consensus sequence and known transcript sequence for two examples, Bt.19462 (Figure 2A) and Bt.17514 (Figure 2B), which aligns to an intronic region of TNFAIP3.

RT-PCR of Mø cDNA with the oligonucleotide pair which spanned CCL5 exon 3 and Bt.19462 amplified four major products, which were cloned and sequenced [GenBank: KP213857–KP213860]. The largest product represented a 1082 nucleotide transcript that extended CCL5 exon 3 through to the Bt.19462 sequence (Figure 2A). The other transcripts had differing 3′ends of exon 3, none of which had the expected splice site motifs, and then included an extra exon which incorporated the Bt.19462 sequence (Figure 2A). The start of this additional exon had the correct splice site motif (AAG|N). All the transcript variants are predicted to result in the generation of the same peptide, with all the variability being in the 3′UTRs.

We were unable to generate a RT-PCR product using oligonucleotides that span exon 1 of TNFAIP3 and Bt.17514. However, oligonucleotides that anneal to Bt.17514 and exon 2 of TNFAIP3 amplified a 777 nucleotide product [GenBank: KP213861]. The first 586 nucleotides match Bt.17514, ending in an appropriate splice site motif (T|GT), and the remaining 191 nucleotides match the start of TNFAIP3 exon 2 (Figure 2B). Further work is required to investigate if Bt.17514 represents an alternative exon 1 for TNFAIP3, which would result in a truncated protein lacking the first 104 amino acids of TNFAIP3. Overall, the results confirm that Bt.17514 and Bt.19462 represent novel splice variants of TNFAIP3 and CCL5 respectively. For functional annotation analysis we have assumed that all unannotated transcripts represent splice variants of neighbouring genes.

Functional annotation clustering analysis using DAVID [23, 24] was carried out to investigate the common transcriptional response of DC and Mø to *Salmonella* stimulation. Only 50% of the genes were assigned GO terms when using the Affymetrix probe-IDs, due to the relative lack of annotation of bovine genes compared to those of human and mouse, which made meaningful functional analysis difficult. Therefore, the HGNC gene symbols of the differentially expressed genes were used in the DAVID analysis, which resulted in the annotation of almost all genes and provided much greater insights into the pathways involved in the common response.

The original DAVID analysis using the bovine gene annotation identified six genes associated with apoptosis and programmed cell death. The reanalysis, using human gene data, identified 16 genes associated with these terms (Table 2). Within these genes there was a bias towards the negative regulation of cell death, with ten of the identified up-regulated genes; BCL2A1, bone marrow stromal cell antigen 2 (BTG2), CFLAR, coagulation factor III (F3), CSF2, immediate early response 3 (IER3), IL6, TNF, TNFAIP3 and vascular endothelial growth factor A (VEGFA), being anti-apoptotic. This suggests that there is a common response of DC and Mø to promote cell survival following stimulation with live and inactivated *Salmonella*. Other GO terms frequently represented in the common response gene list were associated with the regulation of cell communication, with a bias towards GO terms associated with the positive regulation of signalling in cells, e.g., polo-like kinase 2 (PLK2), bone marrow stromal cell antigen 2 (BST2) and CSF2 (Table 2). The involvement of these pathways suggests that there is a common response in DC and Mø to up-regulate the expression of specific transcripts to signal that an event has occurred which requires a response. Other commonly represented GO terms were associated with the immune response and contained several cytokines, e.g., IL6, TNF and chemokines, e.g., CCL5 and CCL20 (Table 2).

**Table 2:** **Clusters of Biological Process GO terms over-represented in the common response gene list**

Biological process GO terms	Genes
*Regulation of cell communication*	
e.g., positive regulation of cell communication positive regulation of signal transduction positive regulation of protein kinase cascade positive regulation of I-κB/NF-κB cascade	BST2, CD80, *CFLAR*, *CSF2*, EDN1, F3, IL6, PLK2, PTGS2, REL, *TNF*, TNFAIP3, VEGFA
*Regulation of programmed cell death*	
e.g., regulation of programmed cell death regulation of apoptosis negative regulation of programmed cell death anti-apoptosis	BCL2A1, BTG2, *CFLAR*, *CSF2*, F3, *GADD45B*, IER3, *IL6*, *NR4A1*, PMAIP1, PTGS2, RYBP, *TNF*, TNFAIP3, TRAF1, VEGFA
*Immune response*	
e.g., inflammatory response immune response response to bacterium chemotaxis	BST2, *CCL5*, *CCL20*, *CSF2*, *CXCL2*, EDN1, F3, *IL6*, KDM6B, MEFV, *NOS2*, PTGS2, RNF19B, *TNF*, VEGFA

Differentially expressed genes (FDR < 0.05, fold change >1.8) identified as common response genes. Genes are listed by HUGO gene symbol and were identified from functional analysis using human gene annotation data. Those indicated in italics were also identified from analysis using bovine gene annotation data.

#### Specific transcriptional responses

Functional annotation clustering analyses of the four differentially expressed gene lists; DC_L, DC_D, Mø_L, Mø_D, using the bovine annotation, illustrated that stimulation with live or inactivated *S.* Typhimurium altered a wide range of cellular processes, including; cell signalling, cell differentiation, cell-cycle and proliferation, transcription and translation, ion homeostasis and metabolism. The analysis revealed that there was considerable overlap in the response of DC and Mø to live and dead *S.* Typhimurium, beyond that suggested by the common transcriptional response gene list. The three most over-represented Biological Process GO term clusters across the gene lists relate to the regulation of programmed cell death, inflammatory response and regulation of transcription. As before, due to the relative paucity of GO annotation of bovine genes a large proportion of the differentially expressed genes were not incorporated in the DAVID analysis. Therefore the data was reanalysed using the human GO annotation. This identified the same three over-represented GO terms, but considerably increased the number of genes associated with them and strengthened the observation that there were differences within these categories associated with cell-type and stimuli. Table 3 summarizes the genes associated with these GO terms identified by functional analysis using bovine and human gene annotations. Almost twice as many genes with the GO term inflammatory response were present in Mø gene lists compared with DC gene lists (Table 3).

**Table 3:** **Summary of genes annotated with the three major GO terms identified from functional analysis of the differentially expressed gene lists**

	DC_L	DC_D	Mø_L	Mø_D
Inflammatory response	*CCL20*, *CCL5*, *CXCL2*, F3, *IL1A, IL1B, IL6*, KDM6B, MEFV, NFKDID, *NFKBIZ*, PTX3, *TNF,* TNFAIP3	*CCL20*, *CCL5*, CCR7, *CXCL2*, F3, *IL6*, KDM6B, MEFV, NFKB1, *OLR1*, *TNF*, TNFAIP3	CD40LG, *CCL20*, *CCL5*, *CCL8*, CCR7, *CXCL2*, *CFB*, F3, IL1RN, *IL1A*, *IL1B*, IL10, *IL6*, KDM6B, MEFV, NFDBID, *NFKBIZ*, *OLR1*, PTX3, *SELP*, *SAA2*, *TNF*, TNFAIP3, TNFAIP6	*CCL20*, *CCL5*, *CCL8*, *CCR4*, CCR7, *CXCL2*, *CFB*, F3, FOS, *IL1A*, *IL1B*, IL10, *IL6*, KDM6B, MEFV, NFKBID, *NFKBIZ*, *OLR1*, PTX3, *SELP*, *SAA2*, *TNF*, TNFAIP3, TNFAIP6
Regulation of programmed cell death	*BCL2A1*, *BIRC3*, BTG2, *CFLAR*, CLCF1, *CSF2*, DUSP1, F3, *GCLC*, IER3, *INHBA*, IFIH1, IL1A, *IL1B*, IL6, NFKBIA, *NR4A1*, *NR4A2*, PMAIP1, *PIM1*, PTGS2, SOCS3, *TNF*, TNFAIP3, TRAF1, TXNIP, VEGFA	*BCL2A1*, *BIRC3*, BTG2, *CFLAR*, *CSF2*, F3, GCH1, *GCLC*, IER3, IL6, NFKB1, NFKBIA, *NR4A1*, *NR4A2*, PPIF, PMAIP1, PIM3, *PLEKHF1*, PTGS2, *TNF*, TNFAIP3, TRAF1, VEGFA	*BCL2A1*, BTG2, CD40LG, *CFLAR*, CLCF1, CD40LG, *CSF2*, DUSP1, F3, GDNF, IER3, *INHBA*, ID3, *IFNG*, IL1A, *IL1B*, *IL10*, IL6, LIG4, LTA, *NR4A1*, PPIF, PMAIP1, *PIM1*, PHLDA1, PTGS2, SOCS3, *TNF*, TNFAIP3, TRAF1, TXNIP, VEGFA	*BCL2A1*, *BIRC3*, BTG2, *CFLAR*, CLCF1, *CSF2*, DLC1, DUSP1, F3, *GCLC*, GDNF, IER3, *INHBA*, ID3, IFIH1, IL1A, *IL1B*, *IL10*, IL6, LIG4, MX1, *NR4A1*, PPIF, PMAIP1, *PIM1*, *PLEKHF1*, PHLDA1, PTGS2, SERPINB2, SOCS3, *TNF*, TNFAIP3, TRAF1, VEGFA
Regulation of transcription	BAZ1A, BHLHE41, BTG2, CD80, *ETS2*, *GCLC*, GZF1, *ID2*, *INHBA*, *IL1B*, *IL6*, *IRF1,* KDM6B, *KLF5*, *NFIL3*, *NFKBIA*, *NFKBIZ*, *NR4A1*, *NR4A2*, NR4A3, PIM1, PNRC1, PRDM1, RYBP, SKIL, *TNF*, TXNIP, VEGFA	BAZ1A, BTG2, *CARHSP1*, CD80, *ETS2*, ETV3, *FOSB*, FOXF1, *GCLC*, GZF1, ICAM1, *IL6*, *IRF1*, *IRF8*, KDM6B, *KLF5*, *MAFF*, *NFATC1*, *NFIL3*, *NFKB1*, *NFKB2*, *NFKBIA*, *NR4A1*, *NR4A2*, NR4A3, PRDM1, RYBP, SAFB2, SFRQ, SKIL, TLE1, *TNF*, VEGFA, ZBTB10	*ATF3*, *BHLHE40*, BHLHE41, BTG2, CD80, *CEBPA*, *CEBPD*, CSRNP1, *EGR1*, EHF, *ETS2*, GDNF, GZF1, *HHEX*, ICAM1, *ID2*, *ID3*, *IFNG*, *IL1B*, *IL10*, *IL6*, *INHBA*, *IRF1*, KDM6B, *KLF13*, *MAFF*, *MED4*, MTERF, *NFATC1*, *NFIL3*, *NFKB2*, *NFKBIZ*, *NR4A1*, PHF10, PPARGC1B, PIM1, PRDM1, RYBP, *TNF*, TXNIP, VEGFA, *ZNF175*, *ZNF181*, ZNF200, *ZNF235*, *ZNF565*, *ZNF567*, *ZNF582*, *ZNF677*, ZNF814	*ATF3*, BAZ1A, BHLHE41, BTG2, CCNL1, CD80, *CEBPD*, CSRNP1, EHF, *ETS2*, ETV3, *FOS*, *FOSB*, *GCLC*, GDNF, *GTF2B*, GZF1, ICAM1, *ID1*, *ID2*, *ID3*, *IL1B*, *IL10*, *IL6*, *INHBA*, *IRF1*, IRF4, KDM6B, *KLF5*, *MAFF*, *NFATC1*, *NFKB2*, *NFKBIZ*, *NR4A1*, PIM1, PRDM1, RYBP, SFPQ, SIK1, SKIL, *TNF*, VEGFA, *ZNF132*, *ZNF582*

Differentially expressed genes (FDR < 0.05, fold change >1.8) identified for DC treated with live (DC_L) and inactivated (DC_D) *S.* Typhimurium and Mø treated with live (Mø _L) and inactivated (Mø _D). Genes are listed by HUGO gene symbol and were identified from functional analysis using human gene annotation data. Those indicated in italics were also identified from analysis using bovine gene annotation data.

The biological process GO terms associated with regulation of programmed cell death also exhibited cell-type differences. The majority of DC_L and DC_D genes assigned to this GO term were associated with the negative regulation of programmed cell death, e.g., 20 out of 31 genes in this cluster are anti-apoptotic. In contrast, the equivalent clusters in Mø gene lists contained a mix of GO terms associated with negative and positive regulation of programmed cell death. For example, the Mø_L gene list contains 18 genes with the GO term Negative regulation of programmed cell death, e.g., CSF2, BTG2, and also 15 genes with the GO term positive regulation of programmed cell death, e.g., inhibitor of DNA binding 3, dominant negative helix-loop-helix protein (ID3). These variations suggest that there are differences, even at early stages of infection, in how DC and Mø respond to stimulation with *S.* Typhimurium.

There are also differences in the Biological Process GO term clusters associated with the regulation of transcription, but these differences are associated with the stimuli and not the cell-type. DC_L and Mø_L genes are biased towards the positive regulation of transcription; for example, the Mø_L gene list contains 19 genes with the GO term positive regulation of transcription and only ten with the GO term negative regulation of transcription. In contrast, DC_D and Mø_D gene lists have more even numbers of negative and positive regulators of transcription. There is an overlap in the genes associated with the GO term negative regulation of transcription in DC_D and Mø_D, including FOSB and ets variant 3 (ETV3). This analysis suggests that at the relatively early time point post stimulation investigated in this study there are differences in the levels of transcription factors which could influence the expression of a large number of genes and thereby modify the response of the cells to live and inactivated *Salmonella*.

### RT-qPCR analysis of differentially expressed genes associated with the GO terms of interest

#### Selection of genes for further analysis

The analysis of the microarray data identified three principal over-represented Biological Process GO terms from the differentially expressed gene lists; inflammatory response, regulation of transcription and regulation of programmed cell death. Intriguingly, these clusters differed with cell-type or stimuli. Therefore we decided to study these pathways in more detail, by investigating the expression of 31 genes, representative of these pathways, in a new set of samples derived from different animals. In addition, the new sample set differed from the previous set in the method used to generate Mø, which resulted in higher levels of cell purity. Furthermore, the *S.* Typhimurium was inactivated with PFA rather than by heat, thus retaining the surface protein structure, which may have been altered by heat and could have affected the detection of bacterial PAMPs by the APC.

The selected genes included several identified by analysis of the microarray data as being common response genes, e.g., chemokine (C-X-C motif) ligand 2 (CXCL2), IL6 and Bt.17514 (TNFAIP3), which are associated with inflammatory response and regulation of programmed cell death GO terms. In addition, genes which were differentially expressed with cell-type were included in the analysis, for example the Mø and DC specific genes ATF3 and NR4A2 respectively. Furthermore, genes exhibiting stimuli-specific differential expression were included, for example TXNIP and FOSB whose expression was only altered in response to live and inactivated *S.* Typhimurium respectively. Genes exhibiting cell-type specific differential expression in the earlier study [11]; CSF2, IL10 and IL12B, were also investigated. As previously mentioned CSF2 is among the common response genes identified in this study and not DC specific as previously found [11], while IL10 was identified as a Mø specific gene in agreement with the earlier study [11]. IL12B was not included in any of the differentially expressed gene lits, although it is represented on the microarray. The expression of several genes, including jun B proto-oncogene (JUNB) and v-myc avian myelocytomatosis viral oncogene homolog (MYC) was also investigated. These genes exhibited differential expression, but below the 1.8-fold cut-off used to generate the differentially expressed gene lists. These were included to ascertain if the criteria used was too stringent.

#### Inflammatory response

The RT-qPCR results for the eight investigated inflammatory response associated genes which were in the common response list; e.g., CXCL2 and MEFV, confirmed that they were up-regulated across all cell and stimulation types. In total thirteen of the 15 investigated inflammatory response genes were up-regulated across all cell and stimulation types (Table 4; IR). The exceptions were IFNG and TXNIP. IFNG was significantly up-regulated in DC in response to live and inactivated *S.* Typhimurium, but only with live bacteria in Mø. TXNIP was only significantly regulated in response to live *S.* Typhimurium in both cell types, in agreement with the microarray data. The microarray data suggested that TXNIP was the most down-regulated gene in response to live *S.* Typhimurium. In contrast, RT-qPCR analysis of the new sample set found that TXNIP expression was up-regulated, by on average 2.2-fold and 3.0-fold for DC and Mø respectively (Table 4). The three putative Mø specific genes identified by analysis of the microarray data; CSF3, IL10 and TNFAIP6, were significantly differentially expressed in Mø and DC. Interestingly CSF2, IL10 and IL12B, which had shown cell-type specific differential expression in a previous study [11], were up-regulated with all cell-types and stimuli.

**Table 4:** **Summary of qRT-PCR results quantifying the expression of genes associated with the three major GO terms investigated in DC and Mø infected with live** ***Salmonella*** **Typhimurium and stimulated with paraformaldehyde-inactivated** ***S.*** **Typhimurium**

Gene symbol	GO term	Average fold changes			
		DC_L	DC_D	Mø_L	Mø_D
ATF3	RT	9.8 ± 1.1^a, b^	6.1 ± 0.5^a, b^	8.5 ± 0.6^a^	6.7 ± 1.2^a^
BIRC3	CD	64.0 ± 15.1^a^	51.0 ± 8.7^a^	81.3 ± 17.1^a^	60.0 ± 9.0^a^
Bt.17514 (TNFAIP3)	IR, CD	53.1 ± 9.8^a^	43.1 ± 5.7^a^	51.8 ± 12.7^a^	59.2 ± 14.3^a^
Bt.19462 (CCL5)	IR, CD	641.0 ± 184.5^a, b^	403.3 ± 90.9^a, b^	443.5 ± 133.6^a, b^	270.9 ± 81.0^a, b^
CSF2	IR, CD	182.0 ± 21.1^a, b^	119.3 ± 26.3^a, b^	257.2 ± 98.5^a^	198.2 ± 78.9^a^
CSF3	IR	308.9 ± 92.0^a^	558.2 ± 243.3^a^	633.8 ± 135.1^a^	548.3 ± 93.2^a^
CXCL2	IR	260.6 ± 38.2^a, b^	147.1 ± 24.4^a, b^	521.6 ± 159.7^a, b^	209.9 ± 46.0^a, b^
ETS1	RT, CD	0.9 ± 0.5	0.5 ± 0.5	0.2 ± 0.6	0.0 ± 0.6
FOSB	RT	3.8 ± 0.9^a, b^	1.5 ± 0.9^b^	4.2 ± 1.1^a, b^	1.6 ± 0.6^a, b^
HHEX	RT	−6.0 ± 1.3^a, b^	−9.9 ± 2.4^a, b^	−8.4 ± 1.0^a^	−9.8 ± 2.5^a^
ID1	RT, CD	71.2 ± 25.6^a^	102.1 ± 39.2^a^	103.0 ± 53.3^a, b^	77.2 ± 42.7^a, b^
ID2	RT	3.1 ± 0.6^a, b ^	1.5 ± 0.6^b^	2.6 ± 0.1^a^	2.2 ± 0.3^a^
ID3	RT, CD	−2.2 ± 0.3^a^	−2.4 ± 0.2^a^	−1.9 ± 0.2^a^	−2.4 ± 0.3^a^
IFNG	IR, CD	589.3 ± 190.5^a, b^	25.1 ± 12.0^a, b^	22.0 ± 9.2^a, b^	2.1 ± 0.8^b^
IL1B	IR, CD	1850.1 ± 415.0^a^	1353.8 ± 333.5^a^	3248.0 ± 1069.1^a^	2048.6 ± 611.3^a^
IL6	IR, CD	79.5 ± 20.7^a^	77.9 ± 31.2^a^	171.1 ± 89.3^a^	97.7 ± 37.9^a^
IL10	IR, CD	69.5 ± 20.4^a^	107.3 ± 31.8^a^	125.7 ± 44.6^a^	112.2 ± 18.6^a^
IL12B	IR, CD	17 044.3 ± 16 476.7^a, b^	34 218.1 ± 32 873.8^a, b^	2591.0 ± 1834.2^a^	3340.4 ± 1966.5^a^
ISG15	IR	304.8 ± 57.8^a, b^	165.7 ± 28.0^a, b^	120.0 ± 48.2^a, b^	46.8 ± 19.0^a, b^
JUNB	RT	15.2 ± 4.1^a, b^	7.4 ± 0.9^a, b^	12.2 ± 1.0^a, b^	8.7 ± 1.0^a, b^
KLF5	RT	9.0 ± 0.8^a^	9.4 ± 1.3^a^	8.7 ± 1.5^a^	9.1 ± 1.4^a^
KLF13	RT	−2.9 ± 0.5^a^	−2.7 ± 0.3^a^	−3.1 ± 0.2^a^	−2.5 ± 0.3^a^
LIG4	RT, CD	2.9 ± 0.3^a^	4.0 ± 0.5^a^	3.2 ± 0.3^a, b^	4.9 ± 0.5^a, b^
MEFV	IR	82.0 ± 27.7^a^	111.6 ± 15.8^a^	76.5 ± 14.3^a, b^	140.1 ± 16.2^a, b^
MYC	RT, CD	−0.5 ± 0.8^b^	−2.3 ± 0.5^a, b^	−0.9 ± 0.6	−1.7 ± 0.2^a^
NR4A2	RT, CD	4.4 ± 0.8^a, b^	1.1 ± 0.5^b^	2.6 ± 0.2^a^	1.9 ± 0.3^a^
PLEKHF1	CD	−5.2 ± 0.7^a^	−4.1 ± 0.6^a^	−3.8 ± 0.6^a^	−3.2 ± 0.3^a^
TNF	IR, CD	353.7 ± 104.3^a^	368.6 ± 58.0^a^	551.6 ± 282.1^a^	452.5 ± 171.5^a^
TNFAIP6	IR	683.3 ± 301.9^a^	927.7 ± 377.9^a^	9115.6 ± 5564.9^a^	9148.4 ± 6115.0^a^
TXNIP	IR, RT, CD	2.2 ± 0.8^a, b^	1.8 ± 0.7^b^	3.0 ± 0.3^a, b^	0.9 ± 0.5^b^
YWHAB	RT, CD	−1.3 ± 0.1^a^	−0.8 ± 0.4	−0.4 ± 0.5	−1.1 ± 0.0^a^

The results are expressed as the average fold change in mRNA levels in DC treated with live (DC_L) and inactivated (DC_D) *S.* Typhimurium and Mø treated with live (Mø _L) and inactivated (Mø _D) *S.* Typhimurium compared to the uninfected DC or Mø ± standard errors.

^a^Indicates when the mean fold change values were significantly different from resting cells by *t* test corrected for multiple testing (Benjamini–Hochberg) (*P* < 0.05).

^b^Denotes when the mean fold change measured in DC and/or Mø is significantly different in the response to live and inactivated *Salmonella* by *t* test corrected for multiple testing (Benjamini–Hochberg) (*P* < 0.05). The GO term column indicates which of the three major GO terms the gene is associated with; inflammatory response (IR), regulation of transcription (RT) and regulation of programmed cell death (CD).

There were no obvious cell-type or stimuli-specific differences in the transcriptional response of Mø and DC with respect to the 15 investigated genes, except TXNIP. However, further statistical analysis identified significant stimuli-specific differences in the expression of eight genes (53%) in the response of DC and/or Mø at the level of fold change (Table 4; b). Six of the investigated genes; Bt.19462 (CCL5), CSF2, CXCL2, IFNG, ISG15 ubiquitin-like modifier (ISG15) and TXNIP, were more highly up-regulated in response to live *S.* Typhimurium than inactivated bacteria, e.g., CXCL2 was up-regulated on average 260.6-fold and 521.6-fold in DC and Mø infected with live *S.* Typhimurium respectively compared with 147.1-fold and 209.9-fold average fold change in DC and Mø respectively stimulated with inactivated *S.* Typhimurium. In contrast, IL12B and MEFV was more highly up-regulated, in DC and Mø respectively, in response to stimulation with inactivated *S.* Typhimurium than live *S.* Typhimurium (Table 4).

**Figure 3:**
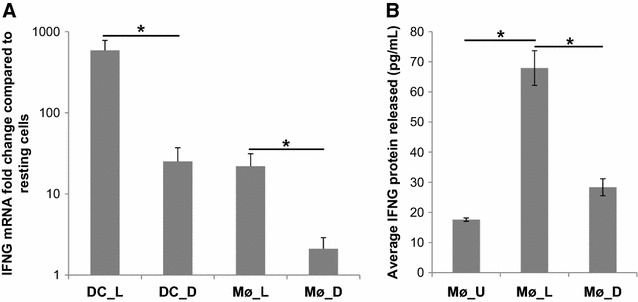
**Production of IFNG by DC and Mø stimulated with live and inactivated** ***S.*** **Typhimurium. A** Average IFNG mRNA levels detected relative to the relevant uninfected sample. Error bars illustrate the standard error of six biological replicates. Asterisk denotes that the variation between live and inactivated *S.* Typhimurium stimulation was statistically different by *t* test (*P* < 0.05). **B** IFNG protein released from unstimulated bovine Mø (Mø_U) and from Mø 8 h post stimulation with live *S.* Typhimurium (Mø_L) and inactivated *S.* Typhimurium (Mø_D). Error bars illustrate the standard error of three biological replicates. Asterisk denotes that the variation between IFNG protein release from Mø infected with live *S.* Typhimurium was significantly higher than that from unstimulated Mø and those stimulated with inactivated *S.* Typhimurium by *t* test (*P* < 0.05).

We were intrigued by the fact that IFNG was identified from the analysis of the microarray data as a gene up-regulated in Mø in response to live *S.* Typhimurium infection. Initially we hypothesized that this resulted from T cell contamination of the Mø samples. However, RT-qPCR analysis confirmed this result in the new samples and showed that IFNG mRNA was up-regulated in Mø and to a greater level in DC in response to live and inactivated *S.* Typhimurium (Figure 3A). There was considerable variation in the level of IFNG mRNA across the biological samples, but there was significantly more produced in response to live *S.* Typhimurium than inactivated bacteria in DC and Mø. IFNG protein in supernatants from Mø stimulated with live and inactivated *S.* Typhimurium for 8 h was measured by ELISA and confirmed that IFNG protein was produced, in response to live *S.* Typhimurium (Figure 3B). The slight increase in IFNG protein secretion observed in response to PFA-treated *S.* Typhimurium was not significantly different from that measured by unstimulated cells (Figure 3B). Since IFNG mRNA levels were not correlated to the level of Mø purity (data not shown), the data strongly suggests that the IFNG is Mø and DC associated.

#### Regulation of transcription

Fifteen genes associated with the regulation of transcription were investigated, the majority of which are transcription factors, e.g., FOSB, hematopoietically expressed homeobox (HHEX). Fourteen of the investigated transcription-associated genes exhibited differential expression in response to live or inactivated *S.* Typhimurium in Mø and/or DC compared to resting cells (Table 4; RT). The exception was ETS1, the expression of which did not significantly alter in either cell-type, in agreement with the microarray data. The four genes, which according to the microarray data analysis exhibited cell-type specific differential expression; ATF3, ID3, ligase IV, DNA, ATP-dependent (LIG4) and NR4A2, were all found to be significantly differentially expressed in both cell types in response to live and inactivated *S.* Typhimurium, except NR4A2 was not significantly differentially expressed in DC stimulated with inactivated *S.* Typhimurium (Table 4).

Several genes exhibited stimuli-specific differential expression in response to live and inactivated *S.* Typhimurium, especially in DC. Five genes; FOSB, ID2, NR4A2, TXNIP and tyrosine 3-monooxygenase/tryptophan 5-monooxygenase activation protein, beta (YWHAB), were differentially expressed in DC in response to live *S.* Typhimurium but not inactivated bacteria. As mentioned above, TXNIP expression also exhibited similar stimuli-specific differential expression in Mø. In addition, live *S.* Typhimurium infection of DC and Mø induced a very variable effect on MYC expression across the biological replicates, whilst stimulation with inactivated *S.* Typhimurium induced down-regulation of MYC, by on average −2.3-fold and −1.7-fold in DC and Mø respectively.

Therefore, although the microarray and RT-qPCR results do not agree for individual genes, the RT-qPCR does support the observation of a stimuli-specific response associated with the regulation of transcription, especially in DC. This was further high-lighted when the statistical analysis of the RT-qPCR data revealed that there were more subtle differences between the response of DC and Mø to live and inactivated *S.* Typhimurium, with ten genes (67%) exhibiting statistically significant stimuli-specific differences in the average fold change values in DC and/or Mø (Table 4; b). In the majority of cases; ATF3, FOSB, ID1, ID2, JUNB, NR4A2 and TXNIP, a greater change in expression was observed in response to live *S.* Typhimurium infection, e.g., JUNB was up-regulated to a higher degree in DC and Mø infected with live *S.* Typhimurium, on average 15.2-fold and 12.2-fold respectively, than in cells stimulated with inactivated *S.* Typhimurium, on average 7.4-fold and 8.7-fold respectively (Table 4). The change in gene expression was greater in response to inactivated *S.* Typhimurium for three genes; LIG4, HHEX and MYC, described above. HHEX was down-regulated to a significantly higher degree in DC in response to inactivated *S.* Typhimurium, on average 9.9-fold, compared to live *S.* Typhimurium, on average 6.0-fold (Table 4). LIG4 was up-regulated to a significantly higher level in Mø in response to inactivated *S.* Typhimurium, on average 4.9-fold, compared to live *S.* Typhimurium, on average 3.2-fold (Table 4).

#### Regulation of programmed cell death

Nineteen genes included in the RT-qPCR analysis have been assigned Biological Process GO terms associated with the regulation of programmed cell death. The majority of the investigated genes are also associated with the over-represented GO terms regulation of transcription, e.g., LIG4, NR4A2, and inflammatory response, e.g., IFNG, IL6. Eight of the investigated genes are associated with the negative regulation of cell death; baculoviral IAP repeat containing 3 (BIRC3), CSF2, ID1, IL1B, LIG4, MYC, NR4A2 and TNFAIP3 and all of these, except MYC, were up-regulated in Mø and DC in response to live and inactivated *S.* Typhimurium, although this did not reach statistical significance for NR4A2 in DC stimulated with inactivated bacteria (Table 4; CD). Four genes; pleckstrin homology domain containing, family F (with FYVE domain) member 1 (PLEKHF1), ID3, IL12B and TXNIP, are associated with the positive regulation of programmed cell death. Interestingly, two of these, PLEKHF1 and ID3, were down-regulated across all cell types and *S.* Typhimurium stimuli, possibly promoting cell survival along with the up-regulation of the negative regulators of cell death. TXNIP, as described previously, was up-regulated in Mø and DC only in response to live *S.* Typhimurium infection, suggesting that the bacteria may be altering the survival state of the infected cell. Overall there is little evidence from the RT-qPCR data to support the cell-type specific difference in the regulation of programmed cell death suggested by analysis of the microarray data. The negative and positive regulators of programmed cell death investigated were similarly expressed in both cell types.

### Further investigation of the transcriptional response of Mø

**Figure 4:**
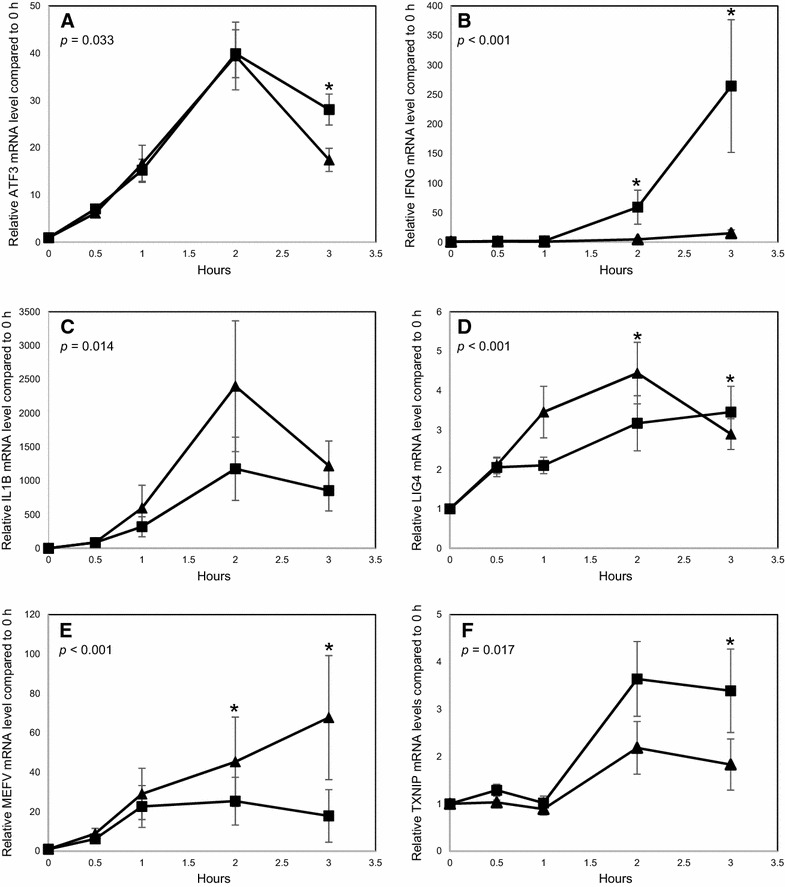
**Transcriptional response of Mø during time course of stimulation with live and inactivated** ***S.*** **Typhimurium.** Average mRNA levels for **A** ATF3, **B** IFNG, **C** IL1B, **D** LIG4, **E** MEFV and **F** TXNIP relative to that measured in the uninfected sample. Closed square and closed triangle denote response to live and PFA-inactivated *S.* Typhimurium respectively. Error bars illustrate the standard error of six biological replicates. *P* values illustrate the significance of stimuli-specific differential expression over the time course. Asterisk denotes if there is a significant difference at each time point (*P* < 0.05).

The observed stimuli and cell-type specific differences in the expression of the investigated genes could be attributed to the design of the microarray experiment and subsequent RT-qPCR analyses which investigated only a single-time point post stimulation. To address this Mø were incubated with live and PFA-inactivated *S.* Typhimurium and the transcriptional response of a subset of the investigated genes was quantified at a range of time points post stimulation. In all six investigated genes, the initial responses to live or inactivated bacteria were indistinguishable (Figure 4), however their expression differed at later time points and all exhibited statistically significant stimuli-specific differential expression across the time course (*P* values shown in graphs). As before, significantly higher levels of IFNG mRNA were detected in response to live *S.* Typhimurium 2 h post infection and also at 3 h (Figure 4B). Similarly, LIG4 mRNA levels were significantly higher in response to inactivated *S.* Typhimurium 2 h post stimulation, however interestingly the reverse was observed at 3 h (Figure 4D). The expression of ATF3 did not differ in response to live or inactivated *S.* Typhimurium at the 2 h time point (Figure 4A), which differs from the previous experiments (Table 4). However, significantly higher levels of ATF3 mRNA were produced in response to live *S.* Typhimurium 3 h post infection. MEFV was more highly expressed in response to inactivated *S.* Typhimurium (Figure 4E), in agreement with previous results (Table 4). This difference was also observed at 3 h. Over the time course IL1B expression was also higher in response to inactivated *S.* Typhimurium (*P* = 0.014), although this difference was not significant at any single time point post infection (Figure 4C). In contrast TXNIP was more highly expressed in Mø in response to live *S.* Typhimurium over the time course (*P* = 0.017), although there was only a significant difference at 3 h post stimulation (Figure 4F). Interestingly, Mø derived from five animals showed an up-regulation of TXNIP, in agreement with our previous RT-qPCR results (Table 4), however Mø from a sixth animal exhibited down-regulation of TXNIP, in agreement with the microarray data, highlighting the difficulty of investigating out-bred biological populations. Overall the results suggest that, at least for the stimuli-specific response observed in Mø, the observed differences were not an artefact of the single time point investigated. The analysis of the selected genes illustrate the divergence of the response of cells to live and inactivated *S.* Typhimurium at a time point when the bacteria is establishing infection.

### Transcriptional differences between DC and Mø

The analysis of the RT-qPCR data described above (Table 4), which calculated the relative fold change within each time course, did not identify many profound differences in the response of DC and Mø to stimulation with *S.* Typhimurium. Therefore, the RT-qPCR data were reanalysed allowing the direct comparison of mRNA levels across both APC and all conditions by calculating the relative fold changes within all samples from each biological replicate. Statistical analysis of these data revealed that 12 (39%) of the investigated genes exhibited cell-type specific differences in average fold-change values, summarized in Table 5. Two genes exhibited differential expression in unstimulated cells. The transcription factor Kruppel-like factor 13 (KLF13) was expressed at significantly higher levels in unstimulated DC and this difference was maintained after stimulation with live and inactivated *S.* Typhimurium. The immune related gene ISG15 was expressed at significantly higher levels in unstimulated Mø, with on average 7.5-fold higher expression in Mø than DC. This differential expression was lost upon stimulation. Six of the genes exhibiting cell-type differential expression upon live and/or inactivated *S.* Typhimurium stimulation were immune related genes and four of these were more highly expressed in Mø than DC, e.g., the pro-inflammatory cytokines IL1B, IL6 and TNF. In contrast, IFNG, which exhibited the greatest cell-type specific differential expression amongst the investigated genes, exhibited on average 15.4-fold greater expression in DC than Mø in response to live *S.* Typhimurium infection. The six transcription factors that exhibited cell-type specific differential expression were evenly separated into those more highly expressed in DC, e.g., KLF13 and those more highly expressed in Mø, e.g., ID3. Furthermore, nine of the genes exhibiting cell-type differential expression are associated with the regulation of programmed cell death, e.g., ID3 and NR4A2, which represents 47% genes associated with this GO term investigated in this study. Overall the analysis of this limited number of genes suggests that the transcriptomes of DC and Mø are similar with respect to the genes that are expressed, but differ in the level of expression of over one-third of those genes, which are associated with all three investigated biological functions; inflammatory response, transcription and programmed cell death.

**Table 5:** **Direct comparison of mRNA levels for investigated genes in DC and Mø**

Gene symbol	Cell-type effect	Average fold differences between cell types					
		Unstimulated cells		Live *S.* Typhimurium		Inactivated *S.* Typhimurium	
CXCL2	*P* = 0.006	Mø	1.1 ± 1.3	Mø	2.7 ± 0.3^a^	Mø	2.0 ± 0.4
ETS1	*P* = 0.023	Mø	1.6 ± 0.6	Mø	1.1 ± 0.4	Mø	1.4 ± 0.6
ID3	*P* < 0.001	Mø	1.7 ± 0.4	Mø	1.6 ± 0.1^a^	Mø	1.3 ± 0.1
IFNG	n.s.	Mø	3.3 ± 0.9	DC	15.4 ± 6.7^a^	DC	4.2 ± 1.3
IL1B	*P* < 0.001	Mø	3.2 ± 1.9	Mø	4.0 ± 1.0^a^	Mø	3.6 ± 0.8^a^
IL6	*P* = 0.019	Mø	8.3 ± 7.4	Mø	7.7 ± 5.5	Mø	8.0 ± 5.0
ISG15	n.s.	Mø	7.5 ± 3.9^a^	DC	1.1 ± 0.2	DC	1.5 ± 0.3
KLF13	*P* < 0.001	DC	1.8 ± 0.2^a^	DC	1.1 ± 0.8^a^	DC	1.7 ± 0.3^a^
NR4A2	*P* = 0.019	DC	1.2 ± 0.1	DC	2.1 ± 0.3^a^	DC	1.0 ± 0.1
TNF	*P* < 0.001	Mø	2.8 ± 1.7	Mø	4.2 ± 1.8^a^	Mø	2.3 ± 0.2
TXNIP	n.s.	DC	0.4 ± 1.4	DC	0.6 ± 0.3	DC	1.2 ± 0.4^a^
YWHAB	*P* = 0.024	Mø	0.2 ± 0.6	Mø	0.1 ± 0.5	Mø	0.4 ± 0.4

The direct comparison the relative mRNA levels in DC and Mø revealed that 12 genes exhibited a cell-type effect by GLM and subsequent Fisher’s test (with Benjamini–Hochberg correction for multiple testing). The cell-type effect column summarizes the statistical significance of the effect of cell-type across all samples by GLM and subsequent Fisher’s test, with Benjamini–Hochberg correction for multiple testing. The average fold difference between the cell types was calculated for each condition (unstimulated, infected with live *S.* Typhimurium, stimulated with inactivated *S.* Typhimurium) and the cell type with the highest level of expression is indicated, with the average fold difference ± standard error.

^a^Indicates where a significant difference in the average fold difference between DC and Mø was identified for the cell type and condition interaction (Fisher’s test, with Benjamini–Hochberg correction for multiple testing *P* < 0.05), n.s. denotes when the effect of cell type was not significant.

### Transcription of the Mediterranean fever gene

**Figure 5:**
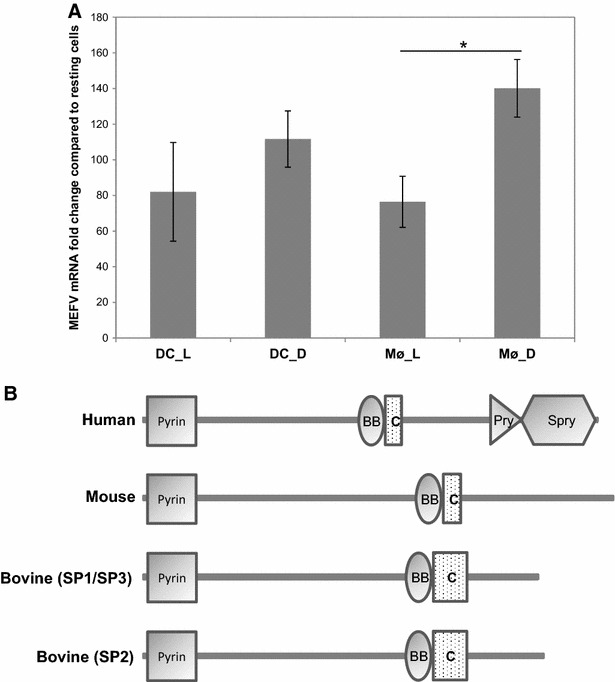
**Investigation and characterization of bovine MEFV.** **A** Average MEFV mRNA levels detected relative to the relevant uninfected sample. Error bars illustrate the standard error of six biological replicates. Asterisk denotes that the variation between live and inactivated *S.* Typhimurium stimulation was statistically different by *t* test (*P* < 0.05). **B** Schematic diagram of human, murine and bovine MEFV proteins illustrating the major protein domains using the web-based SMART tool which identifies protein motifs and architecture [30]. Three splice variants of bovine MEFV were detected; SP1, SP2 and SP3, which are predicted to encode for two protein isoforms. All the proteins contain an N-terminal Pyrin domain as well as B box (BB) and coiled-coil (C) domains. However, only human MEFV encodes a PRY/SPRY (B30.2) domain.

One of the most up-regulated genes, especially in response to inactivated *S.* Typhimurium, identified by analysis of the microarray data was MEFV, which encodes for the protein Pyrin or Marenostrin. RT-qPCR analysis confirmed up-regulation of MEFV in DC and Mø in response to live and inactivated *S.* Typhimurium. The Mø response to inactivated *S.* Typhimurium, with respect to MEFV up-regulation, was significantly greater than that against live *S.* Typhimurium, with on average 140.1-fold and 76.5-fold up-regulation of MEFV mRNA levels in Mø stimulated with live and inactivated bacteria respectively (Figure 5A; Table 4). Mutations in MEFV are associated with the autoinflammatory disease Familial Mediterranean Fever (FMF). MEFV is a multifunctional protein which plays a role in regulating the inflammasome, cytoplasmic multi-protein complexes that, upon activation, process IL1B and IL18 to their mature, active forms. MEFV has previously been shown to be up-regulated in human monocytes and Mø in response to *Burkholderia cenocepacia* infection and plays an important role in regulating IL1B release during infection [29]. Therefore we were interested in the role of MEFV during *S.* Typhimurium infection, since the RT-qPCR results could be explained by *S.* Typhimurium dampening down MEFV expression.

Bovine MEFV had not been characterized previously; therefore we set about generating the full length MEFV transcript sequence for *B. taurus* and *Bos indicus* (Additional file 6). Three splice variants of bovine MEFV were identified [GenBank: JX560181–JX560190], which were all up-regulated in response to stimuli. The three splice variants are predicted to result in two protein isoforms, which differ at the extreme C-terminus (Figure 5B). Analysis of the predicted protein domains using the web-based SMART tool [30] suggests that, similar to murine MEFV, due to frame-shift mutations the bovine orthologue lacks the terminal PRY/SPRY (B30.2) domain of human MEFV (Figure 5B), which contains most mutations associated with FMF. Attempts to detect bovine MEFV protein using four different anti-human MEFV antibodies, all predicted to cross-react with bovine MEFV, failed to detect protein by Western blot (data not shown).

**Figure 6:**
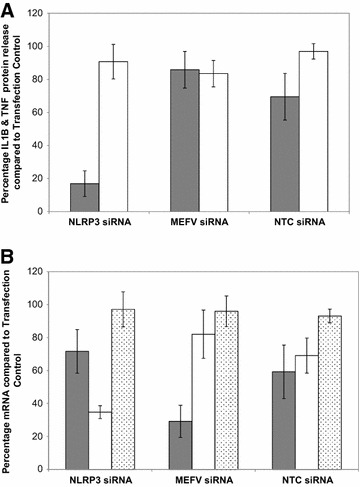
**Investigation of bovine MEFV function.** **A** Quantification of IL1B protein (grey bars) and TNF protein (white bars) released from bovine Mø treated with siRNA targeting NLRP3, MEFV and scrambled siRNA 8 h post infection with live *S.* Typhimurium. **B** RT-qPCR quantification of MEFV (grey bars), NLRP3 (white bars) and IL1B (stippled bars) mRNA levels in bovine Mø treated with siRNA targeting NLRP3, MEFV and scrambled siRNA 8 h post infection with live *S.* Typhimurium. The results are represented as the percentage protein or mRNA compared to the Transfection Reagent only control. Error bars illustrate the standard error of three biological replicates.

The role of bovine MEFV in inflammasome activity in bovine Mø during *S.* Typhimurium infection was investigated using targeted knock-down of all three splice variants using siRNA. In addition, NLRP3, the sensor molecule of the most studied inflammasome, was also targeted by siRNA as a positive control. ELISA analysis of IL1B and TNF, indicators of inflammasome activity and cell activation respectively, protein release 8 h post *S.* Typhimurium infection revealed that NLRP3 knock-down inhibited the release of IL1B, but MEFV knock-down had no effect (Figure 6A). TNF levels were similar across all samples (Figure 6A). RT-qPCR analysis confirmed knock-down of NLRP3 and MEFV (Figure 6B) and knock-down of these genes had no effect of IL1B mRNA levels, confirming that the effect of NLRP3 knock-down was due to loss of inflammasome activity. Similar results were obtained with crude LPS and *B. pseudomallei* infection (data not shown). Recently it has been proposed that MEFV detects the modification of Rho GTPases by bacterial proteins, such as pertussis toxin (PTX) and TcdB cytotoxin from *Bordetella pertussis* and *Clostridium difficile* respectively, and acts as the sensor of a novel inflammasome [31, 32]. However, activation of bovine Mø with PTX and TcdB was not affected by knock-down of MEFV (data not shown). The data suggests that MEFV expression does not influence *S.* Typhimurium infection of bovine Mø and knock-down of MEFV had no effect on *S.* Typhimurium growth (data not shown).

## Discussion

Intestinal Mø and DC play important roles during *S.* Typhimurium infection either as effector cells, removing and destroying invading bacteria, or as activator cells, signalling to other parts of the immune system. However, the ease with which both cell types can take up bacteria also means that these cells provide a safe haven for *S*. Typhimurium and can potentially be hijacked as a Trojan horse. Our present study aimed to extend previous work suggesting that the response of both cell-types to infection with *S.* Typhimurium differed [11], resulting in distinct subsequent inflammatory responses of the gut to *S.* Typhimurium infection.

Analysis of the microarray data revealed that several 100 genes were differentially expressed in DC and/or Mø in response to live and inactivated *S.* Typhimurium. Many of the differentially expressed genes have previously been shown to be modulated during activation or infection of Mø and DC. Interestingly, we identified a common transcriptional response that overlaps with the common response of human Mø to various bacterial pathogens, e.g., up-regulation of IL6, TNF, CSF2, CXCL2, CCL5, interferon regulatory factor 1 (IRF1), ZFP36 ring finger protein (ZFP36), prostaglandin-endoperoxide synthase 2 (PTGS2), ISG15, poliovirus receptor (PVR) and phosphodiesterase 4B, cAMP-specific (PDE4B) [33]. In each gene list approximately 5% of the differentially expressed transcripts were not annotated, having been identified by probes designed from unannotated EST sequences that do not align to exonic sequences, but rather align to intronic or intergenic regions of the current bovine genome assembly. Further analysis of two of these unannotated sequences confirmed that they represent novel transcript variants. The novel transcript of CCL5 exhibited similar patterns and levels of up-regulation across the gene lists as the recognized CCL5 transcript. The second novel sequence investigated was identified as a novel transcript of TNFAIP3, which is not represented by any annotated probe-set on the Affymetrix bovine microarray. Therefore, when analyzing gene lists it is worthwhile investigating and identifying unannotated transcripts, as they can improve knowledge of the transcriptome, especially when investigating species with relatively poor genome annotation.

Functional analysis of the differentially expressed gene lists identified a range of biological processes that were affected by *S.* Typhimurium stimulation. Given their importance for the subsequent immune response, we concentrated on three of these processes; regulation of programmed cell death, inflammatory response and regulation of transcription. These were among the most over-represented terms identified by the analysis and exhibited cell-type and stimuli-specific differences. To investigate these pathways in more detail, we quantified the expression of a selection of representative genes by RT-qPCR, which illustrated the considerable overlap in the response to Mø and DC to live and inactivated *S.* Typhimurium. All the investigated genes were expressed in both cell types, even though several were not identified as such from analysis of the microarray data, and they were similarly modulated in response to stimuli, i.e., were consistently up- or down-regulated in both cell-types. Only more in-depth analysis of the degree of differential expression, i.e., the average fold changes, reveals variation in the response. Therefore, we have focussed on our RT-qPCR analysis to provide insights into the differential response of DC and Mø to live and inactivated *S.* Typhimurium, assuming that the cell-type and stimuli-specific differences observed in the sub-set of investigated genes are indicative of the global response of the cells.

Nearly 40% of the investigated genes were differentially expressed between DC and Mø. The microarray and RT-qPCR analysis revealed that there was a greater inflammatory response in Mø than DC, both in the number of genes differentially expressed as well as in the degree of differential expression observed, in-line with the proposed functions of both cell types. In a recent study comparing the response of porcine monocyte-derived Mø and DC to *S.* Typhimurium infection, Mø again showed a more pro-inflammatory response than DC, e.g., with the production of higher levels of IL1A and IL1B [34]. Furthermore, murine bone-marrow derived Mø produced more IL6 and TNF than bone-marrow derived DC [35]. Therefore, Mø seem to play, at least initially, a major role in alerting and attracting other immune cells to the site of infection. In contrast, we could not verify differences in the expression levels of IL10, CSF2 and IL12B identified in an earlier study [11] as exhibiting cell-type differences in expression during *S.* Typhimurium infection. Given that the methodology of this earlier study was very different than that used in our study, in terms of time-point of analysis as well as MOI of *S.* Typhimurium used [11], this may not be surprising. Indeed, after 24 h stimulation the DC have a more mature phenotype with increased surface expression of CD40, CD80, CD86 and MHC class II [11] and are therefore considerably different to those investigated in our study.

The notable exception to the pattern of higher cytokine expression by Mø than DC was IFNG, which was found to be transcribed at higher levels in bovine DC than Mø. There is now good evidence to support the view that Mø and DC produce IFNG in response to various stimuli [36–38]. IFNG is up-regulated at the mRNA level in bovine Mø stimulated with *Escherichia coli*-derived LPS but not *Mycobacterium bovis*-derived purified protein [39]. Mø were found to be the predominant IFNG producing cell in the spleen during systemic *S.* Typhimurium infection of mice [40]. If the differential transcription of IFNG observed in bovine DC and Mø results in differences in protein secretion, the uptake of *S*. Typhimurium by either cell-type could have a huge impact on the course of infection, as IFNG is a major activator of inflammation, driving direct bacterial killing via oxygen-radical production, enhancing phagocytosis, as well as enhancing antigen presentation [12]. IFNG protein has previously been shown to be produced by human monocyte-derived Mø and DC in response to *S.* Typhimurium infection, albeit at a later time-point of infection [41]. In these experiments production of IFNG by Mø was inhibited by anti-IFNA, anti-IL12 and anti-IL18 antibodies, while IFNG production by DC depended on IL12 and IL18 [41]. In addition, IL12, IL18 and IL27 play an important role in regulating IFNG production by Mø in response to *M. tuberculosis* infection [42]. However, IL12B mRNA levels were significantly higher in DC in response to inactivated *S.* Typhimurium compared to the response to live bacteria, the opposite pattern of IFNG expression. IL27 is not represented on the microarray, while IFNA and IL18, which are represented, were not included in any of the differentially expressed gene lists. Whether these cytokines are involved in the regulation of such early transcription of IFNG in APC requires further investigation.

The comparison of the transcriptional response of Mø and DC by RT-qPCR suggests that it is not the expression of cell-specific genes but the balance of expression of common genes that defines the response of each cell type. This similarity may not be surprising considering that both cell populations are derived in vitro from blood monocytes. Meta-analysis of murine transcriptome data has found that amongst the investigated immune cells bone-marrow derived Mø and DC form a single cluster, which is distinct from classical in vivo isolated DC, e.g., splenic DC [43]. However, there is evidence that the intracellular interactions of *S.* Typhimurium with Mø and DC differ. Mutations in SPI proteins that reduce *S*. Typhimurium survival in Mø do not affect survival in DC [44]. Furthermore, *S*. Typhimurium survives in Mø and DC, but only appears to replicate in Mø [9, 10]. Previous studies have found more profound differences in the response of Mø and DC to *Salmonella* infection, however they have predominantly looked at later time-points when the infection is established and DC have matured [11, 34]. The more subtle changes in gene expression observed in our study, especially those of transcription factors, may account for these later, more obvious, transcriptional differences.

In addition to cell-type differential expression, the RT-qPCR data suggests that the transcriptional response to live and inactivated bacteria differed substantially. Again, in most cases, the response differed in the degree of expression change after stimulation rather than in the expression of a different subset of genes. The average fold change observed in DC and/or Mø in response to live and inactivated *S.* Typhimurium was significantly different in over half the investigated genes (Table 4). These differences were observed in genes associated with all three studied GO terms; however the greatest number was associated with the regulation of transcription. Two-thirds of the investigated transcription factors exhibited stimuli-specific differential expression. Furthermore, in DC one-third of the investigated transcription factors were only differentially expressed in response to live infection. This suggests that either *Salmonella* targets this cellular process, presumably to promote survival, or the host cell recognizes the difference between live and inactivated *Salmonella* and responds accordingly by altering the concentration of transcription factors.

Analyzing the differences in more detail, ATF3 was among the transcription factors whose up-regulation was significantly higher in response to live infection than stimulation with inactivated *Salmonella* in both cell types. Homodimers of this basic leucine zipper transcription factor are negative regulators of inflammation and have been shown to repress the expression of IL6, IL12B and TNF [45]. However, stimuli-specific expression of these genes was not observed in this study. ATF3 dimerises with JUN and FOS family members and the resulting heterodimers can activate or repress the transcription of different genes, e.g., ATF3/JUN dimers enhance the transcription of IFNG in Th1 lymphocytes [46]. The expression of ATF3 is triggered by a wide range of stimuli, including LPS [45] as well as IFNG [47]. ATF3 has previously been shown to be rapidly up-regulated in HeLa cells during *S.* Typhimurium infection due to the activity of SPI-1 effector proteins [48, 49]. Thus, the expression of IFNG and the presence of SPI-1 effector proteins may account for the enhanced expression of ATF3 observed in response to live *S*. Typhimurium infection.

Analysis of the microarray data suggested that a large proportion of the common response genes exhibited average fold increases over 1.5-fold higher in response to inactivated *S.* Typhimurium rather than live bacteria in DC and/or Mø. Four of these genes were included in our RT-qPCR panel; ISG15, Bt.17514 (TNFAIP3), IL6 and MEFV. Of these, a similar pattern of stimuli-specific differential expression was only observed with MEFV in Mø. The pattern of MEFV expression led us to hypothesize that MEFV expression is suppressed by *S.* Typhimurium. MEFV binds to inflammasome components, caspase 1 and PYD and CARD domain containing (PYCARD), and has been postulated to regulate inflammasome activity or act as an inflammasome sensor [32, 50]. Indeed, human, murine and bovine MEFV orthologues all contain the N-terminal Pyrin domain and therefore can interact with inflammasomes via PYCARD. In contrast, bovine MEFV, similar to the murine orthologue, lacks the B30.2 domain present in the MEFV of human and primates. In both murine and bovine MEFV the B30.2 domain has been lost due to frame-shift mutations, which may result from this domain becoming obsolete or it may have been actively lost as the result of adaptation to changes in pathogen exposure. As human MEFV can interact directly with caspase 1 via the B30.2 domain [50], it may play a different role in the function of inflammasomes compared to murine and bovine MEFV. Our experiments using siRNA knock-down failed to confirm a role for bovine MEFV during *S.* Typhimurium infection, in line with previous work investigating IL1B production after *S.* Typhimurium infection of bone marrow-derived Mø derived from *MEFV*^−/−^ mice [32]. In contrast, the knock-down studies show that the NLRP3 inflammasome is functional in bovine Mø during *S.* Typhimurium infection. Interestingly, the NLRP3 inflammasome is inhibited during *S.* Typhimurium infection of murine cells [51], illustrating a clear difference in the response of cells from different mammalian species. Our failure to detect MEFV with four antibodies predicted to cross-react with bovine MEFV by Western blot could imply that the transcripts may not be translated into mature protein, hence the lack of an effect with knock-down. Alternatively, effects on the inflammasome may be an irrelevant read-out to investigate. Human and murine MEFV are multifunctional, interacting with several proteins that affect NF-κB activation, migration, apoptosis and cytoskeletal signalling [50]. However, we did not observe differences in *S.* Typhimurium survival in our MEFV knock-down studies (data not shown). Therefore we conclude that the present data does not support the hypothesis that MEFV expression is specifically targeted by *S.* Typhimurium to promote bacterial survival. The observed stimuli-specific expression may be an off-target effect, due to the bacteria modulating the expression of other genes of importance to the host-pathogen interaction, which are transcriptionally regulated by the same processes as MEFV.

The time course data suggests that stimuli-specific differential expression appears approximately 1–2 h post stimulation (Figure 6), when the *Salmonella* is establishing a niche within SCVs [14]. During the infection of Mø over 900 *S*. Typhimurium genes were found to be differentially expressed, the majority by 4 h post infection [52]. To date over 20 *S.* Typhimurium proteins have been shown to interact with host cell proteins [53]. Several of these bacterial proteins, e.g., SopB and SpvC, can modulate the JNK and MAPK signalling pathways and the activity of NF-κB and AP-1 transcription factors [54–57]. Many of the investigated genes are regulated by these pathways, e.g., NF-κB regulates the expression of many cytokines and regulators of apoptosis [58, 59], which would account for the observed differences in the transcriptional response. However, it is unclear if these effector proteins are acting on the host cell at the time when the stimuli-specific differential expression observed in this study becomes apparent. SPI-2 proteins which form the structure of the T3SS and genes regulating the expression of SPI-2 genes were found to be expressed at relatively high levels 2 h post infection [60], but the secreted effector proteins investigated were maximally expressed later in infection, with relatively low expression at 2 h [60]. Furthermore, SPI-1 protein expression is suppressed in *S.* Typhimurium cultures grown to stationary phase [61], which were used in this study. However, their expression is not totally ablated and levels of several effector proteins increases after phagocytosis [13]. Therefore, although there is limited expression of *Salmonella* effector proteins at the time point investigated, it is possible that these proteins could account for the observed changes of the transcriptional response of DC and Mø to *S.* Typhimurium infection and further investigation involving *S.* Typhimurium mutants is warranted to identify these proteins. Alternatively, the different response of DC and Mø to live and inactivated bacteria may result from the host cell distinguishing between live and dead stimuli and responding accordingly. The observed differences in the investigated subset of differentially expressed genes may have a profound effect on the down-stream response of the infected cell and other cells of the immune response. This may partially account for why killed vaccines are less effective than live vaccines. They elicit poor cell-mediated immunity [62], which may start from the initial response of the phagocytic APC which detects the killed bacteria. Further work is required to elucidate the important aspects of this initial response, modulation of which may enhance the down-stream immune response to vaccines.

In summary, transcriptional analysis of the response of bovine monocyte-derived DC and Mø has revealed a very similar early response by both cell-types to *Salmonella* infection and also to stimulation with inactivated *Salmonella*. However, more in-depth analysis revealed more subtle differences in the intensity of the transcriptional response, which would influence the response of the host cell at later time points and could also modulate the immune response of the infected animal.

## Additional files

10.1186/s13567-016-0328-y **Analysis of bovine monocyte-derived Mø and DC.** A) Flow cytometric analysis of cell purity. B) Flow cytometric analysis comparing the expression of cell-surface markers by monocyte-derived DC and Mø. C) RT-qPCR analysis comparing the mRNA levels of cell-surface markers by monocyte-derived DC and Mø.
10.1186/s13567-016-0328-y **Principal component analysis of microarray data.** Samples are distinguished by A) cell-type; monocyte-derived DC or Mø, B) biological replicate (six digit animal number) and C) condition; uninfected, live *Salmonella* infection or dead *Salmonella* stimulation.
10.1186/s13567-016-0328-y **Table summarizing the oligonucleotides used for the RT-qPCR analysis.** Gene names and symbols are listed with the accession number of the sequence used to design the oligonucleotides. The product size and the sequence of the oligonucleotides is also present in the table.
10.1186/s13567-016-0328-y **Differentially expressed gene lists.** Four Excel sheets listing the genes exhibiting differential expression compared to unstimulated cells in DC infected with live *S.* Typhimurium (DC_L), DC stimulated with inactivated *S.* Typhimurium (DC_D), Mø infected with live *S.* Typhimurium (Mø_L) and Mø stimulated with inactivated *S.* Typhimurium (Mø_D) (FDR ≤ 5%, fold change ≥ 1.8). Genes shown in bold are represented by more than one probe-set. The fold increases and decreases are highlighted in orange and green respectively. Where possible the HGNC gene names and symbols have been used. Transcribed locus denotes an unannotated transcript and their position on the bovine genome relative to neighbouring genes is described.
10.1186/s13567-016-0328-y **An Excel sheet listing the genes forming a common transcriptional response in DC and Mø in response to live and inactivated** ***S.*** **Typhimurium (FDR** **≤** **5%, fold change** **≥** **1.8).** The average fold changes compared to unstimulated cells are listed for each sample set; DC infected with live *S.* Typhimurium (DC_L), DC stimulated with inactivated *S.* Typhimurium (DC_D), Mø infected with live *S.* Typhimurium (Mø_L) and Mø stimulated with inactivated *S.* Typhimurium (Mø_D). Genes shown in bold are represented by more than one probe-set and the average fold change across the probe-sets is listed. Where possible the HGNC gene names and symbols have been used. The list includes five unannotated transcripts and their position on the bovine genome relative to neighbouring genes is described. Fold changes in red highlight where the fold change in DC and/or Mø is more than 1.5-fold different in response to live and inactivated *S.* Typhimurium.
10.1186/s13567-016-0328-y **Description of work to generate MEFV mRNA sequences.** Summary of the methodology used to generate bovine MEFV sequences and the analysis of the sequences.
