# Supplementary material for: Live and inactivated Salmonella enterica serovar Typhimurium stimulate similar but distinct transcriptome profiles in bovine macrophages and dendritic cells
Source: Vet Res. 2016 Mar 22;47:46. doi: 10.1186/s13567-016-0328-y (PMC4802613; doi:10.1186/s13567-016-0328-y)
Supplement: Supplementary file 1 — 10.1186/s13567-016-0328-y Analysis of bovine monocyte-derived Mø and DC. A) Flow cytometric analysis of cell purity. B) Flow cytometric analysis comparing the expression of cell-surface markers by monocyte-derived DC and Mø. C) RT-qPCR analysis comparing the mRNA levels of cell-surface markers by monocyte-derived DC and Mø. [file 13567_2016_328_MOESM1_ESM.docx]

**Phenotypic analysis of monocyte-derived macrophages (Mø) and dendritic cells (DC)**

**Section A: Analysis of Mø and DC purify**

Purified bovine monocyte-derived Mø and DC were analysed by flow cytometry using a mouse anti-bovine SIRPA (CD172α) antibody directly conjugated with RPE-Cy5 (AbD Serotec Cat. No. MCA2041C), a mouse anti-human CD14 antibody directly conjugated with Alexa Fluor® 647 (AbD Serotec Cat. No. MCA1568A647T) and a FACSCaliber (BD Biosciences). In addition, the presence of contaminating T cells and B cells were assessed using a mouse anti-bovine CD3 antibody (Kingfisher Biotech Inc. clone MM1A) and mouse anti-bovine CD21 antibody (AbD Serotec Cat. No. MCA1424GA) respectively with a goat anti-mouse secondary antibody conjugated with RPE (Invitrogen Cat. No. P-21129). The analyses confirmed that the level of purity exceeded 95% in all preparations, with little or no expression of T and B cell markers. Fig. S1 illustrates the flow cytometry results for Mø and DC from an example animal.


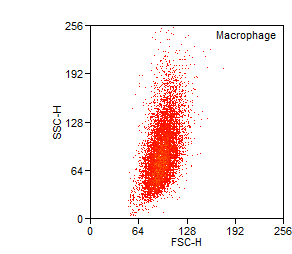

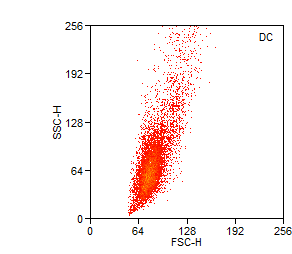


Macrophage

DC

**A**

**C**

**B**

**D**

Macrophage

DC

**E**

**F**

Fig. S1. Flow cytometry analysis of bovine monocyte-derived Mø and DC. A & C, scatterplots illustrating the size (FSC) and granularity (SSC) of Mø and DC populations respectively. B & D, histograms illustrating the level of surface expressed CD14 (blue) and SIRPA (green) compared to unstained cells (red) in Mø and DC respectively. E & F, histograms illustrating the level of surface expressed CD3 (green) and CD21 (blue) compared to isotype control (red) in Mø and DC respectively. The data shown are for cells generated from one representative animal.

**Section B: Expression of surface markers**

To compare the phenotypes of the generated bovine monocyte-derived Mø and DC, flow cytometric analysis of a selection of myeloid cell-surface markers was undertaken. Primary antibodies, described in Table S1, were used in conjunction with a goat anti-mouse IgG1 secondary antibody conjugated with RPE (Invitrogen Cat. No. P-21129). All the flow cytometry analysis was carried out using a FACSCaliber (BD Biosciences).

The seven investigated cell-surface molecules were expressed on both bovine monocyte-derived Mø and DC. The results for Mø and DC generated from a representative animal are illustrated in Fig. S2. The mean fluorescence intensities (MFI) of five molecules; CD1B, CD11B, CD11C, CD86 and major histocompatibility complex (MHC) class II DR were consistently higher on DC than Mø in all the investigated cell preparations. The greatest difference in cell surface expression was observed with CD1B and MHC class II DR, where the MFI was more than two fold greater for DC than Mø. The surface expression of CD40 and CD80 did not exhibit cell type differential expression across all the cell preparations. The results show that the culture conditions employed; adherence and high FBS concentration for Mø and the presence of IL4 and CSF2 for DC, have induced the bovine monocytes to differentiate into distinct cell populations.

Table S1. Monoclonal antibodies used for flow cytometric analysis.

|  |  |  |  |  |
| --- | --- | --- | --- | --- |
| Antigen | Clone | Isotype | Source | Cat. No. |
|  |  |  |  |  |
|  |  |  |  |  |
| CD1B | CC14 | IgG1 | AbD Serotec | MCA831G |
| CD11B | CC94 | IgG1 | IAH | [65] |
| CD11C | NAM4 | IgG1 | IAH | [65] |
| CD40 | IL-A156 | IgG1 | AbD Serotec | MCA2431GA |
| CD80 | IL-A159 | IgG1 | AbD Serotec | MCA2436GA |
| CD86 | IL-A190 | IgG1 | AbD Serotec | MCA2437GA |
| MHC class II DR | CC108 | IgG1 | AbD Serotec | MCA5656 |
| Isotype control | AV20 | IgG1 | IAH | [65] |
|  |  |  |  |  |
|  |  |  |  |  |

IAH, Institute for Animal Health

[65] Corripio-Miyar *et al.* (2015) Veterinary Research 46:112

DC

CD1B

Macrophage

CD1B

Macrophage

MHCII

DC

MHCII

Macrophage

CD11B

DC

CD11B

Macrophage

CD11C

DC

CD11C

DC

CD40

DC

CD80

DC

CD86

Macrophage

CD40

Macrophage

CD80

Macrophage

CD86

Fig. S2. Flow cytometric analysis of surface molecule expression on bovine monocyte-derived Mø and DC. The results are represented as histograms illustrating the expression of investigated molecules (green lines) compared to isotype control (red lines). The cell type and surface molecule are indicated on each histogram. The data shown are for cells generated from one representative animal.

**Section C: mRNA levels of DC & Mø markers**

To further phenotype the generated bovine monocyte-derived Mø and DC, RT-qPCR analysis was carried out to quantify the mRNA levels of CD1B and CD180. The RT-qPCR analysis was carried out as described in the manuscript Materials & Methods using pre-determined optimal concentrations of oligonucleotides for CD1B (5’- AGGTGGTGACAATGAGGATG-3’ and 5’- GCCTGAGCCTTGATTTTGAG-3’) and CD180 (5’- CATCGTCCACATTTCAGTGC-3’ and 5’- GGAAACTGGCAGCATTGATT-3’).

Fig. S3 illustrates that there was significant cell-type differential expression of CD1B (Fig. S3.A), with on average 7 fold higher mRNA levels in DC than Mø, in agreement with the surface expression results (Fig. S2). Conversely, CD180 expression was on average 2 fold higher in Mø than DC (Fig. S3.B). Furthermore, recently the transcriptomes of bovine monocyte-derived Mø and DC were compared by RNASeq analysis and thousands of genes were found to be significantly differentially expressed between the two cell types (Glass, unpublished data). These data provide further evidence that the culture methods employed have generated distinct cell populations.


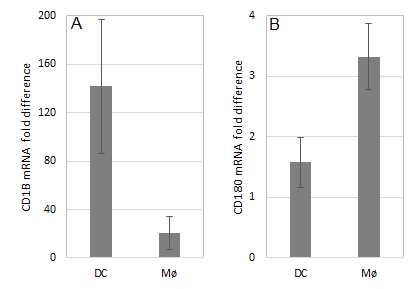


Fig. S3. Surface molecule mRNA levels in bovine monocyte-derived DC and Mø cell preparations. Average A) CD1B and B) CD180 mRNA levels detected in DC and Mø cell preparations compared to the sample exhibiting the least expression. Error bars illustrate the standard error of the mean of six biological replicates.
