# Supplementary figures and images for: Live and inactivated Salmonella enterica serovar Typhimurium stimulate similar but distinct transcriptome profiles in bovine macrophages and dendritic cells
Source: Vet Res. 2016 Mar 22;47:46. doi: 10.1186/s13567-016-0328-y (PMC4802613; doi:10.1186/s13567-016-0328-y)

## Slide 1
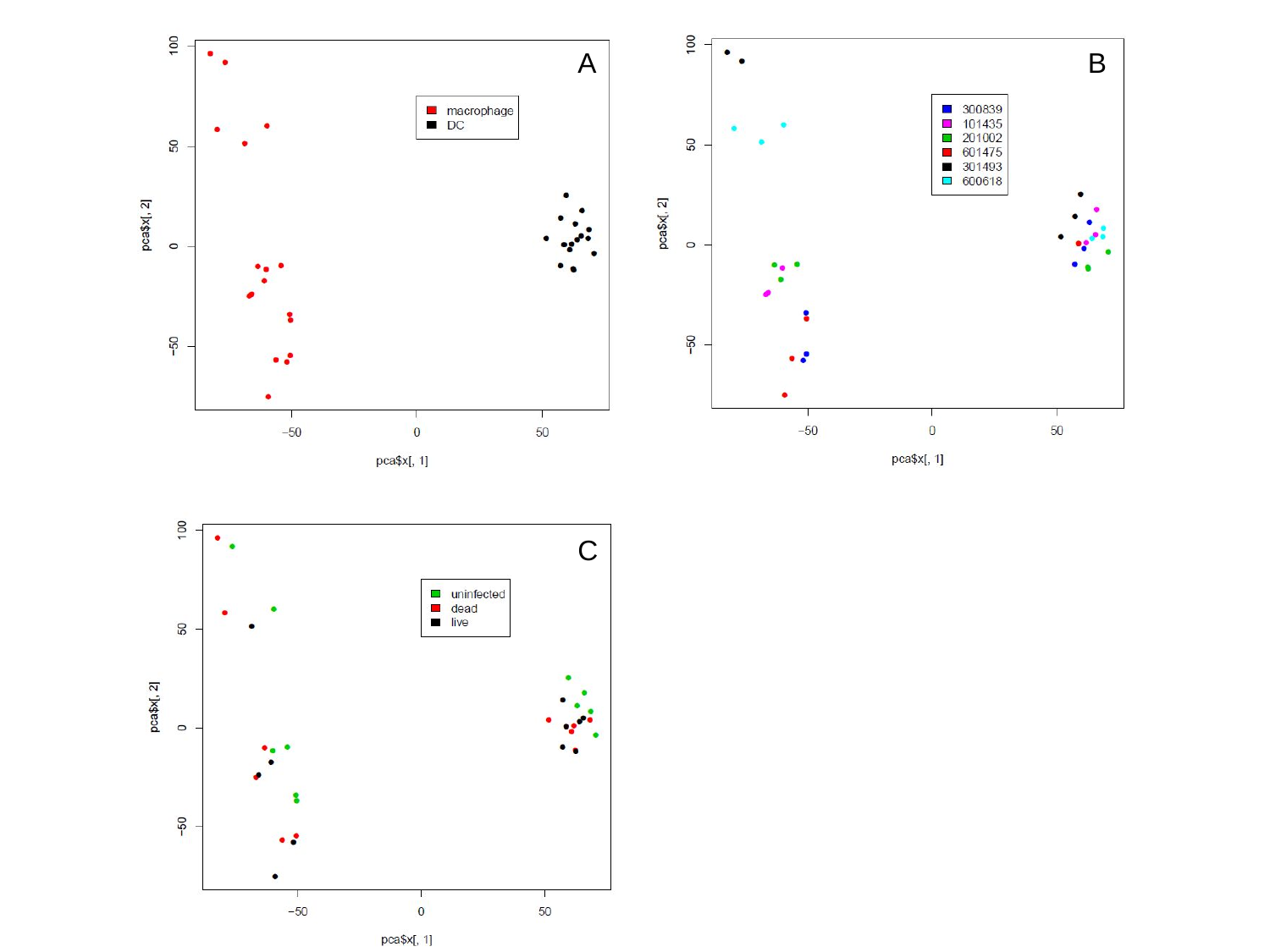

A
B
C

Supplement: Supplementary file 2 — 10.1186/s13567-016-0328-y Principal component analysis of microarray data. Samples are distinguished by A) cell-type; monocyte-derived DC or Mø, B) biological replicate (six digit animal number) and C) condition; uninfected, live Salmonella infection or dead Salmonella stimulation. [file 13567_2016_328_MOESM2_ESM.pptx]
