# Supplementary material for: Live and inactivated Salmonella enterica serovar Typhimurium stimulate similar but distinct transcriptome profiles in bovine macrophages and dendritic cells
Source: Vet Res. 2016 Mar 22;47:46. doi: 10.1186/s13567-016-0328-y (PMC4802613; doi:10.1186/s13567-016-0328-y)
Supplement: Supplementary file 3 — 10.1186/s13567-016-0328-y Table summarizing the oligonucleotides used for the RT-qPCR analysis. Gene names and symbols are listed with the accession number of the sequence used to design the oligonucleotides. The product size and the sequence of the oligonucleotides is also present in the table. [file 13567_2016_328_MOESM3_ESM.docx]

|  |  |  |  |  |  |
| --- | --- | --- | --- | --- | --- |
| **Gene** | **Gene**  **Symbol** | **Accession**  **Number** | **Forward primer (5’-3’)** | **Reverse primer (5’-3’)** | **Size (bp)** |
|  |  |  |  |  |  |
|  |  |  |  |  |  |
| activating transcription factor 3 | ATF3 | NM_001046193 | ATGTCTTCCGCTTGGCTCT | CGTGTTTCTGCTTTCTTCCTG | 86 |
| baculoviral IAP repeat containing 3 | BIRC3 | NM_001035293 | ACAGTTGAGGAGACTACAAGAAGAA | CACAAGGAATAAACACTATGGACAC | 80 |
| Affymetrix probeset Bt.17514 | Bt.17514 | - | TGAGGAAGGGAGTAGGTGGT | CAAGTGGGTGTGGTCAGG | 126 |
| Affymetrix probeset Bt.19462 | Bt.19462 | - | GGGCAAGTTATTTCCATCTCC | ATCCACCATCCTTCCTCCTC | 102 |
| colony stimulating factor 2 (GM-CSF) | CSF2 | NM_174027 | CAGCCCAGAAGTGAAGCAG | GTGGTCCCTCCAGTGTGAA | 118 |
| colony stimulating factor 3 (G-CSF) | CSF3 | NM_174028 | CCTTCACTTCAGCCTTCCAA | CCTTTCTGTGCTTCCCTGTC | 202 |
| chemokine (C-X-C motif) ligand 2 | CXCL2 | NM_174299 | CAAGAACATCCAGAGCGTGA | GGGTTGAGACACACTTCCTGA | 102 |
| v-ets erythroblastosis virus E26 oncogene homolog 1 (avian) | ETS1 | NM_001099106 | ACAAGCCCGTCATTCCTG | ATTCCCAGCCATCTCCTGTC | 138 |
| FBJ murine osteosarcoma viral oncogene homolog B | FOSB | NM_001102248 | CCTCCCTTCTTGCTCTGTGA | CCCTCTCTCCTCCAGTTCCT | 89 |
| hematopoietically expressed homeobox | HHEX | NM_001105424 | GTTCTCCAATGACCAGACCA | TTGACCTGCCTCTCGCTAA | 117 |
| inhibitor of DNA binding 1, dominant negative helix-loop-helix protein | ID1 | NM_001097568 | GCACTCTCAACGGCGAAAT | TTCAGCGACACAGGATACGA | 81 |
| inhibitor of DNA binding 2, dominant negative helix-loop-helix protein | ID2 | NM_001034231 | TCAACACCGACATCAGCATC | CACCATTTATTCAGCCACAGAG | 99 |
| inhibitor of DNA binding 3, dominant negative helix-loop-helix protein | ID3 | NM_001014950 | CAGCACTTCCCAACCTCATT | GTTCCGACAGGCAGCATAC | 85 |
| interferon gamma | IFNG | NM_174086 | GCAAGTCTATGGGATTTCAAGG | GGCATCATTTCATTTATCAGCA | 127 |
| interleukin 1, beta | IL1B | NM_174093 | TCCGACGAGTTTCTGTGTGA | TGTGAGAGGAGGTGGAGAGC | 122 |
| interleukin 6 | IL6 | NM_173923 | ACCACTCCAGCCACAAACAC | ATGCCCAGGAACTACCACAA | 176 |
| interleukin 10 | IL10 | NM_174088 | TGGATGACTTTAAGGGTTAC | AGGGCAGAAAGCGATGAC | 183 |
| interleukin 12B (p40) | IL12B | NM_174356 | GCCTGCTTATTGAGGTCGTG | AGGTTCTTGGGTGGGTCTG | 109 |
| ISG15 ubiquitin-like modifier | ISG15 | NM_174366 | GATCAATGTGCCTGCTTTCC | TCAGCCACAGTCTGCTTCAG | 204 |
| jun B proto-oncogene | JUNB | NM_001075656 | GCCCTTCTACCACGACGACT | AGGCTGGGTTTCAGGAGTTT | 96 |
| Kruppel-like factor 5 (intestinal) | KLF5 | NM_001083727 | CGAATCTGGAATGCTTGCTA | GCTTTGGGAAGAACACTGC | 122 |
| Kruppel-like factor 13 | KLF13 | NM_001083533 | TTCTCGTGATTCCGTGTCTTT | TGTTGTTGTTGTTGTCGTCGT | 92 |
| ligase IV, DNA, ATP-dependent | LIG4 | NM_001191126 | CAGGAATACGACCAATACGG | CCAAATCAGCAATCACAGGA | 133 |
| Mediterranean fever | MEFV | XM_002706315 | CCAGGAAGGAAAGAAACAAAG | TGGAGAAATGGTAAGTTCAAGAC | 87 |
| v-myc myelocytomatosis viral oncogene homolog (avian) | MYC | NM_001046074 | CTCTCTGCTCTCCTCTGCTG | TTCCTCATCCTCTTGTTCTTCC | 118 |
| NLR family, pyrin domain containing 3 | NLRP3 | NM_001102219 | GTTGTCCGTTTCCTCTTTGG | AGTGTCTTGGCATTGGCTTT | 137 |
| nuclear receptor subfamily 4, group A, member 2 | NR4A2 | NM_001076208 | CAAAGCCGACCAAGACCT | CCAAAGCCACGAACACATT | 147 |
| pleckstrin homology domain containing, family F (with FYVE domain) member 1 | PLEKHF1 | NM_001082468 | AGGGACAAGAGAGAAGGTAGGAC | AGCAGAGTGGGTGGGTAGGT | 144 |
| squamous cell carcinoma antigen recognized by T cells | SART1 | NM_001193086 | ATGAAGAAGCTGGACGAGGA | GGAGGGATCAGAAGGGAGAC | 190 |
| tumour necrosis factor | TNF | NM_173966 | GGGACACCCAGAATGTGAG | ATTGGCAGGAAGGGAGAGTT | 115 |
| tumour necrosis factor, alpha-induced protein 6 | TNFAIP6 | NM_001007813 | CAATAGAAGTGAAAGATGGGATGC | ATTTGGGAAGCCTGGAGATT | 109 |
| thioredoxin interacting protein | TXNIP | NM_001101875 | TTGATTCTGAGGCGATGTCC | CCCTGTCATTGTGCTTACCC | 116 |
| tyrosine 3-monooxygenase/tryptophan 5-monooxygenase activation protein, beta polypeptide | YWHAB | NM_174794 | ATGGGAGAGTCAGGAATAGCC | GCAGATGGACAAACAGATGG | 100 |
|  |  |  |  |  |  |
