# Supplementary material for: Live and inactivated Salmonella enterica serovar Typhimurium stimulate similar but distinct transcriptome profiles in bovine macrophages and dendritic cells
Source: Vet Res. 2016 Mar 22;47:46. doi: 10.1186/s13567-016-0328-y (PMC4802613; doi:10.1186/s13567-016-0328-y)
Supplement: Supplementary file 6 — 10.1186/s13567-016-0328-y Description of work to generate MEFV mRNA sequences. Summary of the methodology used to generate bovine MEFV sequences and the analysis of the sequences. [file 13567_2016_328_MOESM6_ESM.docx]

**Generation of Mediterranean fever (MEFV) mRNA sequences**

**Methodology**

Monocytes were isolated from Holstein-Friesian (*Bos taurus*) and Sahiwal (*Bos indicus*) cattle as described previously [66], except that the monocytes were selected using anti-human CD14 conjugated microbeads (Miltenyi Biotec). In addition, monocyte-derived macrophages (Mø) were generated as described previously [17]. RNA from resting and crude LPS activated bovine monocytes and Mø was reverse transcribed using GoScript (Promega) according to the manufacturer’s instructions, with either oligo(dT) primer or Primer PR1 (Table 1). MEFV transcripts were amplified with ABgene Taq Polymerase (Thermo Scientific) following the manufacturer’s instructions and using a combination of forward and reverse primers listed in Table 1, which had been designed using Primer3 [25, 26] and the predicted MEFV sequence available at that time [GenBank:XM_002706315.1]. This predicted sequence has subsequently been removed from the database and replaced with a record which incorporates the sequences generated in this study. In addition, 3’RACE products were generated using MEFV specific forward primers and Primer PR2. The resulting PCR products were purified using QIAquick Gel Extraction Kit (Qiagen) following the manufacturer’s instructions. Purified PCR fragments were cloned into pGEM-T easy vector (Promega) and the resulting plasmids purified using the Qiaprep miniprep kit (Qiagen) following the manufacturers’ instructions. The purified plasmids were sequenced using Big Dye Sequencing and T7 and SP6 oligonucleotides by Edinburgh Genomics. The resulting sequences were combined to generate virtually full length sequences, which were submitted to GenBank [GenBank: JX560181-JX561190].

**Results**

The majority of primer combinations resulted in a single product. However, sets amplifying the region containing nucleotides 1400-2100 generated multiple products, suggesting that there were MEFV splice variants. This was confirmed by analysis of the sequenced fragments and identified three major splice variants. All three splice variants were sequenced for Holstein-Friesians. Due to technical difficulties sequence was only generated for the two most abundant splice variants from Sahiwal-derived monocytes. The resulting ten sequences have been submitted to Genbank [GenBank: JX560181 - JX560190]. Aligning the MEFV sequences to the bovine genome reveals that all three splice variants have simpler gene architecture than the predicted sequence XM_002706315.1, consisting of 9 or 10 exons (Fig. 1). Splice variant 2 (MEFV_SP2) uses a different start site for exon 9 than splice variant 1 (MEFV_SP1), resulting in a shorter transcript. Splice variant 3 (MEFV_SP3) uses the same start site for exon 9 as MEFV_SP1, but the exon is truncated and there is a tenth exon, which utilizes the same start site as MEFV_SP2 exon 9.

The generated sequences suggest that the three MEFV splice variants encode truncated proteins, all encoding stop codons within exon 9. MEFV_SP1 and MEFV_SP3 have the same stop codon, which is the last codon of MEFV_SP3 exon 9, whilst MEFV_SP2 skips this sequence but has a down-stream stop codon near the start of its exon 9 (Fig. 1).

**Table 1. Summary of MEFV and 3’RACE primers used to amplify MEFV transcript fragments.**

|  |  |  |  |
| --- | --- | --- | --- |
| Oligonucleotide | Orientation | Sequence 5’-3’ | Position |
|  |  |  |  |
|  |  |  |  |
| Pyrin_1_forward | F | TCTTCTGGTCACCACCATGG | 17-36 |
| Pyrin_1_reverse | R | CTTTTGGGTCGCTTCTTTCC | 805-786 |
| Pyrin_2_forward | F | GGAAAGAAGCGACCCAAAAG | 786-805 |
| Pyrin_2_reverse | R | GCTTGCTTCATGTGACGCT | 1426-1408 |
| Pyrin_3_forward | F | AAGCGTCACATGAAGCAAGC | 1407-1426 |
| Pyrin_3_reverse | R | AGTGGACAGTTTCTGCTTCC | 2106-2087 |
| Pyrin_4_forward | F | GGAAGCAGAAACTGTCCACT | 2087-2106 |
| Pyrin_4_reverse | R | TTATGAGGTCCAGCCACAGT | 2879-2860 |
| MEFV02 | R | AGGAGCCTGAAGGGACATG | 2476-2458 |
| MEFV03 | F | CGTGAAGCACTTCTTGGAGA | 1991-2010 |
| MEFV04 | F | CTGCGCTCAGAAGTGGAAAC | 2013-2032 |
| PR1 | R | GGCCACGCGTCGACTAGTACTTTTTTTTTTTTTTTTT | - |
| PR2 | R | GGCCACGCGTCGACTAGTAC | - |
|  |  |  |  |

F & R denote forward and reverse oligonucleotides respectively. Position summarizes which nucleotides the oligonucleotides anneal to in predicted bovine MEFV sequence (Accession No. XM_002706315.1).


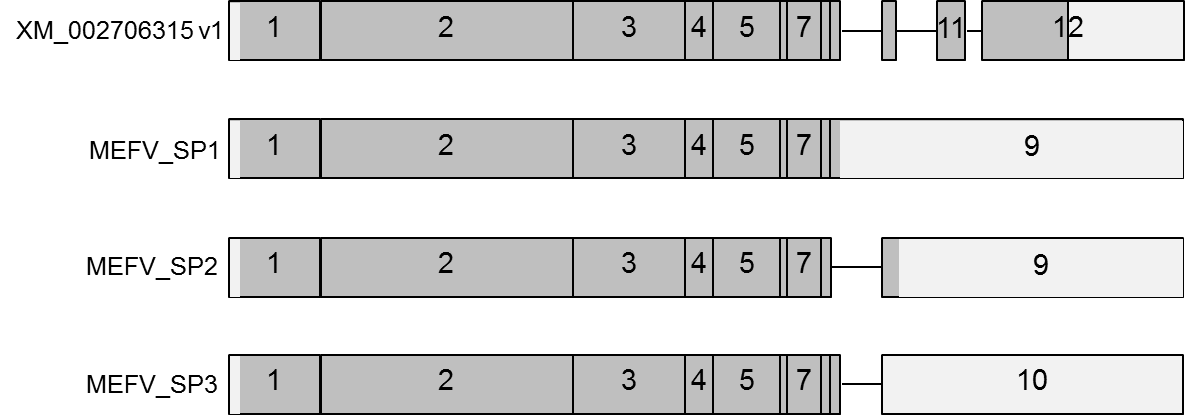


Figure 1. Gene architecture of the sequenced bovine MEFV splice variants. The gene architecture of the three identified splice variants: MEFV_SP1, MEFV_SP2 and MEFV_SP3 is simpler than that predicted from the RefSeq sequence XM_002706315.1. The exons are shown as boxes and the introns are not shown, except to highlight differences between the gene architectures. The numbers denote the exon number and the dark grey shading illustrates the predicted translated sequence. All three bovine splice variants encode long 3’UTRs.

**References**

[17] Jensen K *et al.,* 2009. Int. J. Parasitol. 39:1099-1108.

[25] Koressaar T & Remm M, 2007. Bioinformatics 23:1289-1291.

[26] Untergrasser V *et al.,* 2012. Nucleic Acids Res 40:e115.

[66] Jensen K *et al.*, 2014. Vet. Immunol. Immunopathol. 158:224-232.
